# Selecting PedsQL items to derive the PedsUtil health state classification system to measure health utilities in children

**DOI:** 10.1186/s12955-024-02268-5

**Published:** 2024-07-10

**Authors:** Ellen Kim DeLuca, Kim Dalziel, Eve Wittenberg, Nicholas C. Henderson, Lisa A. Prosser

**Affiliations:** 1 Department of Population Medicine, Harvard Pilgrim Health Care Institute, Boston, MA USA; 2 Department of Health Management and Policy, Michigan School of Public Health, University of Michigan, Ann Arbor, MI USA; 3 Centre for Health Policy, Melbourne School of Population and Global Health, University of Melbourne, Parkville, VIC Australia; 4 Center for Health Decision Science, Harvard T.H. Chan School of Public Health, Harvard University, Boston, MA USA; 5 Department of Biostatistics, Michigan School of Public Health, University of Michigan, Ann Arbor, MI USA; 6 Susan B. Meister Child Health Evaluation & Research Center, Department of Pediatrics, Michigan Medicine, University of Michigan, Ann Arbor, MI USA

**Keywords:** Health-related quality of life, PedsQL, Pediatrics, Rasch analysis

## Abstract

**Background:**

There is a lack of preference-based health-related quality of life (HRQoL) measures that consistently value health across a full range of child age groups. The PedsQL is a generic HRQoL instrument validated for children 2–18 years, but it is not preference-based. The objective of this study was to derive the PedsUtil health state classification system from the PedsQL as a basis for a preference-based HRQoL measure for children.

**Methods:**

A two-step process was used to select PedsQL items to include in the health state classification system: 1) exclude poorly functioning items according to Rasch analysis in each of the previously established seven dimensions of the PedsUtil health state classification system and 2) select a single item to represent each dimension based on Rasch and psychometric analyses, as well as input from child health experts and parents. All secondary analyses were conducted using data from the Longitudinal Study of Australian Children (LSAC). Analyses were stratified by age group (i.e., 2–5 years, 6–13 years, and 14–17 years) to represent the different developmental stages of children and to reflect the study design of the LSAC. Rasch analyses were also performed on five random subsamples for each age group to enhance robustness of results.

**Results:**

Twelve items were excluded from the PedsUtil health state classification system after the first step of the item selection process. An additional four items were excluded in the second step, resulting in seven items that were selected to represent the seven dimensions of the PedsUtil health state classification system: Physical Functioning (“participating in sports activity or exercise”), Pain (“having hurts or aches”), Fatigue (“low energy level”), Emotional Functioning (“worrying about what will happen to them”), Social Functioning (“other kids not wanting to be their friend”), School Functioning (“keeping up with schoolwork”), and School Absence (“missing school because of not feeling well”).

**Conclusions:**

The PedsUtil health state classification system was derived from the PedsQL based on several criteria and was constructed to be applicable to children two years and older. Research is ongoing to elicit preferences for the PedsUtil health state classification system to construct the PedsUtil scoring system.

**Supplementary Information:**

The online version contains supplementary material available at 10.1186/s12955-024-02268-5.

## Background

An important methodological challenge in conducting economic evaluations in child health is the estimation of health utilities to derive quality-adjusted life years, the standard health outcome measure used in cost-effectiveness analyses, for pediatric populations. Commonly used generic preference-based measures of health-related quality of life (HRQoL), such as the EQ-5D [1], SF-6D [2] or HUI-3 [3], were primarily developed for adults. There are some child-specific preference-based HRQoL measures, such as the CHU-9D [4], EQ-5D-Y [5] and HUI-2 [6], but many were primarily developed for children five years and older. Given the desire to consistently measure HRQoL across childhood, some of these child-specific instruments are now adapting approaches and validating measurement in younger children [7–9]. However, preference-based scoring systems are currently lacking for younger age groups in some of these child-specific measures. In addition, the approaches that have been used to value these child-specific measures are highly variable across measures, and sometimes even within the same measure for valuations completed in different countries [10–14]. Therefore, further research is required to design and produce preference-based HRQoL measures that can be consistently applied across a wide range of pediatric age groups.

One method to derive a preference-based HRQoL measure is to develop a health utility scoring system for an existing non-preference-based measure. The Pediatric Quality of Life Inventory (PedsQL) is a generic, non-preference-based HRQoL instrument that is validated for children 2–18 years [15, 16]. The PedsQL has a long tradition of use in clinical trials for pediatric interventions. Providing a health utility scoring system for the PedsQL, the PedsUtil scoring system, will allow for economic endpoints to be estimated directly from the PedsQL without the need for additional resource-intensive data collection. The PedsUtil scoring system can be constructed by first developing a health state classification system (HSCS) based on the PedsQL and then by obtaining preference weights for the HSCS. Previous studies have adopted modern psychometric approaches to construct a HSCS from an existing non-preference-based measure [17–21], such as the development of the SF-6D from the SF-36 [2, 22]. This paper applies and adapts these previously used methods to derive the PedsUtil HSCS. The objective of this study was to utilize Rasch analysis alongside other psychometric methods and expert and parent opinion to select a subset of PedsQL items to construct the PedsUtil HSCS.

## Methods

### The PedsQL

The PedsQL 4.0 Generic Core Scales is a validated instrument that assesses HRQoL across four dimensions: 1) Physical Functioning (8 items), 2) Emotional Functioning (5 items), 3) Social Functioning (5 items), and 4) School Functioning (3–5 items depending on age group) (Appendix Table 1) [15, 16]. Both child self-report (5–18 years) and parent proxy-report (2–18 years) versions are available. The items in the different versions are very similar and differ only in developmentally appropriate vocabulary and first- or third-person tense. For each item, respondents are asked to choose from a series of five severity levels: 0 = Never, 1 = Almost never, 2 = Sometimes, 3 = Often, 4 = Almost always. Level responses are converted to non-preference-based HRQoL scores and can be reported in terms of domain scores, a Physical Health Summary Score, Psychosocial Health Summary Score, and overall Total Score [16].

### Overview of analysis

**Fig. 1:**
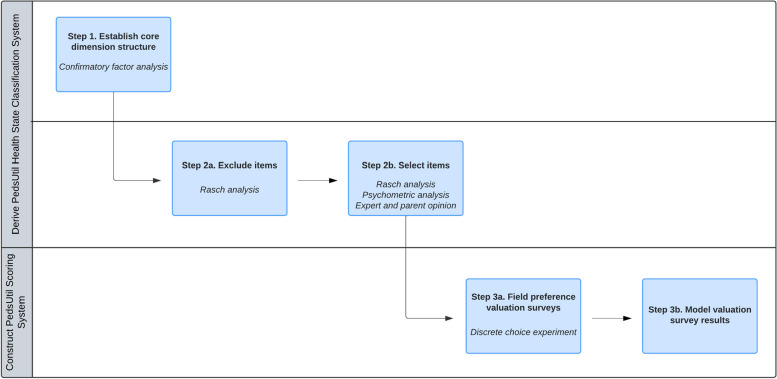
Steps to Constructing the PedsUtil Health State Classification System and Scoring System

With 23 items, each ranging five severity levels from “Never” to “Almost always”, the PedsQL defines 5^23^ unique health states. It is necessary to reduce the length of the PedsQL to construct a HSCS that is feasible for preference valuation methods. One useful technique to help inform which items to include or exclude from a HSCS is Rasch analysis [23]. Rasch analysis can be used to evaluate measurement functioning and psychometric properties of existing instruments by providing empirical evidence on how well items in a dimension measure the construct of interest (e.g., physical functioning) [24, 25]. In this study, a two-step process was used to select items to include in each dimension of the PedsUtil HSCS (Fig. 1). The first step was to exclude any poorly functioning items in each dimension by examining various Rasch criteria. The second step was to then select a single item to represent each dimension among the remaining items based on Rasch and other psychometric criteria, as well as input from child health experts and parents. This study was granted an exempt determination by the University of Michigan Institutional Review Board (IRBMED # HUM00182088).

### Data source

All secondary analyses were conducted using data from the Longitudinal Study of Australian Children (LSAC), a national-level population-representative study that collects data from 10,000 children and families every two years [26]. The LSAC delivers a comprehensive dataset on the development of children over time and is one of the very few large-scale nationally representative studies of children in the world. A nationally representative sample, which includes a wide spectrum of healthy and unwell children, was used for data analysis to ensure that the resulting HSCS can be applied to such populations. The LSAC sampling design is detailed elsewhere [27]. The LSAC was approved by the Australian Institute of Family Studies Ethics Committee, and families provided written informed consent to participate [28].

This study used data from the first seven waves (2003–04 to 2015–16) of the LSAC (*n* = 45,207) (Appendix Table 2). This dataset contains fully completed responses to the parent proxy-report version of the PedsQL at each wave of data collection for the same children at different ages from 2–17 years with the exception that the LSAC only administered 19 out of 21 PedsQL items for children aged 2–3 years (the two items on school absence were omitted). Consequently, only 19 PedsQL items were included in the dataset for children aged 2–3 years. This dataset also included information on child special healthcare needs status (yes/no) defined as “a condition which has lasted or is expected to last for at least 12 months, which causes the child to use medicine prescribed by a doctor, other than vitamins, or use more medical care, mental health or educational services” [29]. Child special healthcare needs status was determined for each child using data from the last available wave since younger children are less likely to be identified as having special healthcare needs because not enough time may have passed for their symptoms to have fully manifested or been recognized.

### Data analysis – confirmatory factor analysis

Prior to item selection, the dimension structure of the PedsUtil HSCS must be established. Confirmatory factor analysis was previously conducted using data from the LSAC to establish this core dimension structure; technical details of this analysis are reported elsewhere [30]. The findings from this study supported a 7-dimension structure of the PedsUtil HSCS: 1) Physical Functioning (6 items); 2) Pain (1 item); 3) Fatigue (1 item); 4) Emotional Functioning (5 items); 5) Social Functioning (5 items); 6) School Functioning (3 items); and 7) School Absence (2 items). Following dimension identification, a single item was selected to represent each dimension of the HSCS using the methods described below; single-item dimensions (i.e., Pain and Fatigue) were not empirically evaluated in the item selection process as they were already represented by one item.

### Data analysis – Step 1: item exclusion

The purpose of the first step in the item selection process was to eliminate unsuitable items based on their poor psychometric performance. Data were fitted to the Rasch partial credit model to test how well the observed data meet expectations of the measurement model. If there was any misfit, adjustments were made until a well-fitting model was achieved, but items that exhibited misfit were considered for exclusion. Since Rasch models assume unidimensionality, a separate model was estimated for each multi-item dimension using RUMM2030 [31]. Analyses were stratified by age group (i.e., 2–5 years, 6–13 years, and 14–17 years) to select items that would be applicable across a wide range of ages. These specific age groupings were selected to represent the different developmental stages of children, as well as to reflect the study design of the LSAC. Three main Rasch criteria were used to assess item performance and are briefly described below. Refer to Appendix A for more details of each criterion.

#### Item level ordering

Item-threshold probability curves were first examined to determine if disordering was present [32]. For items that exhibited disordered thresholds, ordering of items was achieved by collapsing adjacent item response levels. If there was more than one possible combination to merge levels, the combination that demonstrated the best overall fit while also achieving a more balanced distribution across levels was selected. Disordered items were evaluated for exclusion as they failed to respond to the full range of severity across the dimension.

#### Differential item functioning (DIF)

Once all items were ordered, DIF by sex and child special healthcare needs status was examined since the PedsUtil HSCS needs to apply across diverse pediatric populations. Both uniform and nonuniform DIF were tested for using analysis of variance [33]. Items exhibiting DIF were separated into different person factors and the Rasch model was refit. If splitting the item did not improve model fit, the item was considered for removal from the Rasch model. Items exhibiting DIF were assessed for exclusion as they threaten construct validity and are of limited value for making cross-population comparisons.

#### Rasch model goodness-of-fit

After issues of disordered thresholds and DIF were resolved, overall model fit was assessed by examining the item-trait interaction statistic, reported as a ${\chi }^{2}$ statistic. If overall model fit was poor (i.e., *p*-value < 0.01 with a Bonferroni correction), the fit of the individual items was examined. Items with fit residuals greater than the standard cutoff $\pm$ 2.5 and with statistically significant individual ${\chi }^{2}$ statistics were dropped from the model sequentially, beginning with the worst fitting item [32]. This procedure was repeated until only well-fitting items remained and the overall item-trait interaction statistic was nonsignificant. Items that were dropped from the Rasch model poorly represent the underlying dimension being measured, thus were considered for exclusion.

#### Robustness check

In order to enhance robustness, Rasch analysis was conducted on five subsamples of the LSAC dataset for each age group for a total of 15 subsamples. Stratified random sampling was used to obtain subsamples of approximately 500 responses, which is the recommended sample size for Rasch analysis [34]. Sampling was stratified on child sex, age, and special healthcare needs status (Appendix Table 2). Each item per age group was given a total score (out of five) indicating the number of subsamples that the item performed well on all Rasch criteria. In general, any item that performed poorly across all five subsamples in any age group (i.e., score of 0/5) or was the worst fitting item in any age group (i.e., lowest total score) was excluded from the PedsUtil HSCS.

### Data analysis – Step 2: item selection

Following Step 1, a single best item was selected for each dimension among the remaining items. A range of criteria (described below) was considered for item selection.

#### Rasch analysis

Individual item goodness-of-fit statistics were assessed, and the item with the better fit to the Rasch model was generally considered to be the better item to represent the dimension. The spread of item thresholds was also examined. An item that covers a wider severity range was considered to better represent the dimension than an item that covers a narrow range.

#### Other psychometric criteria

Internal consistency (i.e., correlation of an item score and its dimension score) and floor and ceiling effects were examined. Items with low correlation were considered to not be representative of the dimension and items exhibiting large floor or ceiling effects were regarded to be poor candidates as they may poorly respond to the full severity range of the dimension. These criteria were evaluated in relative terms between items as done in previous studies [18, 19, 21] rather than applying strict thresholds.

#### Expert and parent opinion

Expert and parent opinion were collected to supplement Rasch and psychometric analyses as statistical analyses alone may not be able to identify the single best item for each dimension. Moreover, stakeholder engagement was used to assess content and face validity of the PedsUtil HSCS. Previous studies have similarly engaged with various stakeholders to aid in item selection [20, 35].

A US-based convenience sample of five pediatricians and one clinical trialist were recruited to provide input on item selection for all age groups, and 12 parents were recruited to provide input on item selection for each age group of their children. The clinicians included general pediatricians and specialists. The parents included parents of children with special healthcare needs (e.g., diabetes, asthma, musculoskeletal conditions, depression, anxiety, and ADHD) and of typically functioning children from ages 2–17 years (Appendix Table 3). Participants were asked to select which item best represents each dimension of the PedsUtil HSCS and to provide justifications for their choices. Refer to Appendix B for more details.

#### Final item selection

The research team evaluated results from all criteria to make the final decisions for item selection. The final PedsUtil HSCS was reviewed with an external health status measurement expert to ensure that the items selected were cohesive and amenable to preference valuation methods required to construct the PedsUtil scoring system.

## Results

### Step 1 – Item Exclusion

Table 1 displays the total scores indicating how many subsamples each item performed well on all Rasch criteria (i.e., item ordering, DIF, and item fit). Appendix Tables 4A-4F provide more detailed results.

**Table 1:** Summary of Item Performance on Rasch Criteria for Item Exclusion

**Item Description ***Problems with…*	**Total Score**^a^			**Item Excluded**
	**2–5 years**	**6–13 years**	**14–17 years**	
**Physical Functioning**^b^				
Phys1. Walking	0/3^c^	1/5	1/3^c^	**✔**
Phys2. Running	0/3	4/5	3/3	
Phys3. Participating in exercise	0/3	4/5	3/3	
Phys4. Lifting something heavy^d^	–	–	–	**✔**
Phys5. Taking a bath or shower^d^	–	–	–	**✔**
Phys6. Doing chores^d^	–	–	–	**✔**
**Emotional Functioning**				
Emot1. Feeling afraid or scared	4/5	2/5	2/5	
Emot2. Feeling sad or blue	1/5	5/5	0/5	**✔**
Emot3. Feeling angry	4/5	1/5	1/5	
Emot4. Trouble sleeping	2/5	0/5	2/5	**✔**
Emot5. Worrying	4/5	5/5	5/5	
**Social Functioning**				
Soc1. Getting along with others	0/5	0/5	0/5	**✔**
Soc2. Others not wanting to be friends	4/5	4/5	3/5	
Soc3. Getting teased	4/5	1/5	2/5	
Soc4. Unable to do things others can do	1/5	0/5	0/5	**✔**
Soc5. Keeping up with other children	0/5	0/5	0/5	**✔**
**School Functioning**				
School1. Paying attention in class	–^e^	0/5	2/5	**✔**
School2. Forgetting things	–^e^	0/5	0/5	**✔**
School3. Keeping up with schoolwork	Only item^f^	0/5	4/5	
**School Absence**				
SchAbs1. Missing school because sick	2/5^g^	4/5	3/5	
SchAbs2. Missing school to go to doctor	0/5^g^	0/5	0/5	**✔**

*Abbreviations*: *Emot* Emotional Functioning, *Phys* Physical Functioning, *SchAbs* School Absence, *School* School Functioning, *Soc* Social Functioning

^a^Total score = number of subsamples item performed well on all Rasch criteria (out of five subsamples)

^b^Most subsamples did not fit the Rasch model for the Physical Functioning dimension, thus supplemental Rasch analyses were conducted for items Phys1-Phys3. Results from the supplemental analyses are shown in the table

^c^Insufficient sample size to obtain five subsamples so only three subsamples were created for supplemental analyses (total score out of three)

^d^Items Phys4-Phys6 were omitted from the supplemental Rasch analyses, thus total scores were not calculated for these items. Refer to Results section for more details

^e^School1 and School2 are not included in the PedsQL for children under 5 years old

^f^Only School3 is included in the PedsQL for this age group

^g^SchAbs1 and SchAbs2 were not administered for children aged 2–3 years in the LSAC so results reflect responses for children aged 4–5 years

#### Physical functioning

Four out of five subsamples did not fit the Rasch model for age groups 2–5 years and 6–13 years (i.e., item-trait interaction ${\chi }^{2}$ statistic was statistically significant). For the subsample that did fit the Rasch model, only Phys3 (“participating in exercise”) performed well on all Rasch criteria (Appendix Table 4A). For age group 14–17 years, all five subsamples fit the Rasch model, but Phys1 (“walking”), Phys5 (“taking a bath or shower”), and Phys6 (“doing chores”) scored 0/5. Because most of the subsamples misfit the Rasch model, supplemental Rasch analyses were performed on items Phys1-Phys3, which the research team considered the most relevant items in this dimension (Appendix Table 4B). As a result, items Phys4-Phys6 were excluded from the PedsUtil HSCS. For age group 2–5 years, none of the supplemental subsamples fit the Rasch model, thus results from the other age groups were used to help guide item exclusion. For age groups 6–13 years and 14–17 years, Phys1 was the worst performing item (total score 1/5 and 1/3, respectively), thus was excluded.

#### Emotional functioning

Emot2 (“feeling sad or blue”) was the worst performing item for age groups 2–5 years (total score 1/5) and 14–17 years (total score 0/5), thus was excluded (Appendix Table 4C). For age group 6–13 years, Emot4 (“trouble sleeping”) exhibited disordered thresholds and/or item misfit in all subsamples (total score 0/5), thus was also excluded.

#### Social functioning

Soc1 (“getting along with others”) and Soc5 (“keeping up with other children”) scored 0/5 for all age groups and Soc4 (“unable to do things others can do”) scored 0/5 for age groups 6–13 years and 14–17 years (Appendix Table 4D). Therefore, these three items were excluded.

#### School functioning

Since School Functioning consisted of only one item for age group 2–5 years, Rasch analysis was not conducted for this age group. For age group 6–13 years, Rasch analysis provided little insight for item exclusion as none of the items performed well in any of the subsamples. School1 (“paying attention in class”) and School3 (“keeping up with schoolwork”) exhibited disordered thresholds and DIF and School2 (“forgetting things”) did not fit the Rasch model (Appendix Table 4E). Consequently, Rasch analysis for age group 14–17 years was primarily used to help guide item exclusion across all ages. For age group 14–17 years, School2 did not fit the Rasch model in any of the subsamples, thus was excluded. School1 was also excluded at this point because School1 is not a validated PedsQL item for children under five years old, and the HSCS needs to apply across all age groups. In addition, School1 (total score 2/5) performed worse than School3 (total score 4/5) for age group 14–17 years.

#### School absence

Though none of the subsamples could be fitted to the overall Rasch model, SchAbs2 (“missing school to go to doctor”) performed worse across all age groups (total score 0/5) than SchAbs1 (“missing school because sick”) (Appendix Table 4F). The individual ${\chi }^{2}$ statistics for SchAbs2 were statistically significant for all subsamples, indicating poor item fit to the Rasch model. Therefore, SchAbs2 was excluded.

### Step 2 – Item selection

Table 2 provides a summary of results for the remaining nine items following Step 1. Appendix Table 6 also includes an item-by-item summary of performance and details when items were excluded and which were selected.

**Table 2:** Summary of Criteria for Item Selection for Remaining Items (Additional Information in Appendix Tables 4A-5E)

**Item Description** *Problems with…*	**Age Group**	**Rasch Criteria**^b^							**Other Psychometric Criteria**^a,b^			**Expert and Parent Opinion**^b^	
		**Total Score**^c^	**Mean Item Level Performance**^d^ (Range Across Subsamples)			**Disordered Thresholds**	**DIF**^e^	**Item Misfit**	**% Response Ceiling (Never)**	**% Response Floor (Almost always)**	**Int. Consistency**^f^	**Best Item – Experts**^g^	**Best Item – Parents**^h^
			***p*-value**^i^	**Fit Residual**^j^	**Spread**								
**Physical Functioning**^k^													
Phys2. Running	2-5y	0/3^l^	–	–	–	2 samples	No DIF	3 samples	91.8%	0.3%	0.62^m^	0/6	1/6
	6-13y	4/5	0.08 (0.004, 0.13)	1.13 (1.09, 1.17)	0.68 (0.64, 0.72)	None	None	1 sample	76.0%	1.8%	0.82	0/6	0/6
	14-17y	3/3^l^	0.06 (0.02, 0.09)	0.70 (0.11, 1.10)	0.63 (0.56, 0.78)	None	No DIF	None	70.6%	2.3%	0.82	0/6	0/3
Phys3. Participating in exercise	2-5y	0/3^l^	–	–	–	3 samples	No DIF	3 samples	80.5%	0.6%	0.67^n^	5/6	5/6
	6-13y	4/5	0.26 (0.06, 0.63)	0.42 (0.08, 1.16)	0.59 (0.54, 0.64)	1 sample	None	1 sample	73.1%	3.1%	0.86	5/6	6/6
	14-17y	3/3^l^	0.05 (0.02, 0.13)	0.37 (0.002, 0.57)	0.61 (0.50, 0.72)	None	No DIF	None	67.7%	3.5%	0.87	5/6	3/3
**Emotional Functioning**													
Emot1. Feeling afraid or scared	2-5y	4/5	0.16 (0.05, 0.40)	0.90 (0.30, 1.38)	1.10 (0.99, 1.28)	1 sample	No DIF	No misfit	23.0%	0.3%	0.72	3/6	4/6
	6-13y	2/5	0.27 (0.08, 0.45)	1.13 (0.86, 1.41)	1.09 (0.96, 1.22)	1 sample	2 samples (Sex)	No misfit	35.8%	0.5%	0.77	0/6	1/6
	14-17y	2/5	0.06 (0.005, 0.12)	1.23 (0.85, 1.62)	0.94 (0.76, 1.12)	1 sample	2 samples (Sex) 1 sample (SHCN status^o^ and Sex)	No misfit	53.0%	0.5%	0.78	0/6	0/3
Emot3. Feeling angry	2-5y	4/5	0.51 (0.09, 0.84)	0.72 (0.26, 1.72)	1.57 (1.24, 1.97)	1 sample	1 sample (Sex)	No misfit	12.2%	0.2%	0.67	0/6	0/6
	6-13y	1/5	0.25^p^	1.13	0.97	None	3 samples (Sex) 1 sample (SHCN status and Sex)	4 samples	15.4%	0.7%	0.69	0/6	0/6
	14-17y	1/5	0.57^p^	1.50	1.08	None	3 samples (Sex)	1 sample	19.0%	1.0%	0.75	0/6	0/3
Emot5. Worrying	2-5y	4/5	0.05 (0.004, 0.11)	1.26 (0.03, 1.81)	0.99 (0.76, 1.28)	None	No DIF	1 sample	50.9%	0.2%	0.72	1/6	2/6
	6-13y	5/5	0.25 (0.04, 0.51)	0.52 (0.38, 0.74)	0.98 (0.75, 1.32)	None	No DIF	No misfit	39.7%	0.9%	0.77	1/6	3/6
	14-17y	5/5	0.33 (0.01, 0.68)	1.20 (0.47, 1.69)	0.99 (0.81, 1.12)	None	No DIF	No misfit	36.6%	1.1%	0.81	1/6	2/3
**Social Functioning**													
Soc2. Others not wanting to be friends	2-5y	4/5	0.11 (0.01, 0.36)	1.28 (0.18, 2.23)	1.14 (0.88, 1.34)	1 sample	No DIF	No misfit	46.8%	0.2%	0.75	2/6	3/6
	6-13y	4/5	0.24 (0.02, 0.90)	1.17 (0.42, 1.64)	1.25 (0.75, 1.84)	1 sample	1 sample (Sex)	No misfit	43.4%	0.9%	0.78	2/6	1/6
	14-17y	3/5	0.17 (0.09, 0.27)	0.65 (0.46, 1.0)	1.04 (0.76, 1.35)	1 sample	2 samples (Sex)	No misfit	51.4%	0.6%	0.80	2/6	1/3
Soc3. Getting teased	2-5y	4/5	0.10 (0.01, 0.27)	1.05 (0.63, 2.15)	1.02 (0.81, 1.18)	1 sample	No DIF	No misfit	60.8%	0.1%	0.68	0/6	0/6
	6-13y	1/5	0.22^p^	0.31	0.92	3 samples	1 sample (SHCN status)	No misfit	42.4%	0.7%	0.76	0/6	0/6
	14-17y	2/5	0.57 (0.44, 0.70)	0.43 (0.01, 0.86)	0.87 (0.77, 0.97)	2 samples	1 sample (Sex)	No misfit	54.0%	0.6%	0.77	0/6	0/3
**School Functioning**													
School3. Keeping up with schoolwork	2-5y	Only item included – Rasch analysis not performed^q^							62.5%	2.6%	N/A	6/6	6/6
	6-13y	0/5	–	–	–	3 samples	5 samples (Sex)	No misfit	39.2%	5.0%	0.89	6/6	6/6
	14-17y	4/5	0.83 (0.76, 0.87)	0.44 (0.34, 0.60)	1.13 (1.08, 1.22)	1 sample	1 sample (Sex)	No misfit	26.5%	5.4%	0.89	6/6	3/3
**School Absence**													
SchAbs1. Missing school because sick	2-5y^r^	2/5	0.15 (0.10, 0.19)	0.37 (0.17, 0.57)	2.08 (1.86, 2.30)	1 sample	2 samples (SHCN status)	No misfit	58.2%	0.1%	0.91	6/6	6/6
	6-13y	4/5	0.06 (0.01, 0.15)	0.53 (0.33, 0.73)	1.67 (1.10, 2.03)	None	No DIF	1 sample	47.0%	0.3%	0.90	6/6	6/6
	14-17y	3/5	0.04 (0.02, 0.06)	0.09 (0.02, 0.16)	1.22 (1.14, 1.32)	None	No DIF	2 samples	38.3%	1.2%	0.91	6/6	3/3

*Abbreviations*: *DIF* differential item functioning, *Emot* Emotional Functioning, *Int*. *Consistency* internal consistency, *N/A* not applicable, *Phys* Physical Functioning, *SCHN* special healthcare needs, *SchAbs* School Absence, *School* School Functioning, *Soc* Social Functioning, *y* years

^a^Psychometric criteria were assessed using the full LSAC sample (*n* = 45,207) rather than using just the Rasch analysis subsamples

^b^Results from Rasch analyses for all items shown in Appendix Tables 4A-4F and psychometric analysis and expert and parent opinion for all items shown in Appendix Tables 5A-5E

^c^Total score = number of subsamples item performed well on all Rasch criteria (out of five subsamples)

^d^Mean item level performance calculated using only the subsamples that the item performed well on all Rasch criteria

^e^A *p*-value < 0.05 (with Bonferroni adjustment) was used to determine presence of DIF

^f^Correlation of item score with dimension score for dimensions identified for the PedsUtil HSCS (i.e., Spearman’s correlation coefficient)

^g^Number of experts (out of *n* = 6) that chose an item as the best item to represent this dimension

^h^Number of parents that chose an item as the best item to represent this dimension. There were *n* = 6 parents with children 2–5 years old, *n* = 6 parents with children 6–13 years old, and *n* = 3 parents for children 14–17 years old (*n* = 12 parents but some parents had children in different age groups so total adds up to more than 12)

^i^*p*-value for the individual item ${\chi }^{2}$ statistics

^j^Fit residuals may be positive or negative, thus absolute value of the fit residuals reported in the table

^k^Results from supplemental Rasch analyses shown in table

^l^Insufficient sample size to obtain five subsamples so only three subsamples were created for supplemental analyses

^m^Correlation of Phys2 with Physical Functioning dimension if the dimension included only three items (Phys1-Phys3) was 0.86 for age group 2–5 years, 0.93 for age group 6–13 years, and 0.92 for age group 14–17 years

^n^Correlation of Phys3 with Physical Functioning dimension if the dimension included only three items (Phys1-Phys3) was 0.83 for age group 2–5 years, 0.91 for age group 6–13 years, and 0.91 for age group 14–17 years

^o^Special healthcare needs status defined as children with special healthcare needs or typically functioning children

^p^Only one subsample performed well on all Rasch criteria, thus no range reported in table

^q^School3 is only item included in the PedsQL for this dimension for this age group, thus Rasch analysis was not performed

^r^School Absence items were not asked for children aged 2–3 years in the LSAC, thus results reflect responses for children aged 4–5 years

#### Physical functioning

Phys2 (“running”) and Phys3 (“participating in exercise”) remained in this dimension following Step 1. Both items similarly fit the Rasch model and had similar item spread (Appendix Table 4B). Both demonstrated large ceiling effects (≥ 67.7%), though they were less severe for Phys3 (Appendix Table 5A). Both items also had high internal consistency across age groups, but correlations were higher for Phys3 (0.67–0.87). Most experts (5/6) and parents (11/12) thought Phys3 was the best item to represent the dimension. Therefore, Phys3 was selected for the PedsUtil HSCS.

#### Emotional functioning

Among the remaining items (Emot1, Emot3, and Emot5), Emot3 (“feeling angry”) was the worst performing item based on Rasch criteria (total score 1/5 for age groups 6–13 years and 14–17 years) and had the lowest internal consistency (Appendix Tables 4C and 5B), thus was excluded. Between Emot1 (“feeling afraid or scared”) and Emot5 (“worrying”), Emot5 had higher total scores across all age groups. However, Emot1 had larger item spread for age groups 2–5 years and 6–13 years, while Emot5 had larger item spread for age group 14–17 years. Emot5 exhibited large ceiling effects (51%) for age group 2–4 years, while Emot1 exhibited large ceiling effects (53%) for age group 14–17 years. Emot1 was chosen most often by experts (3/6) as the best item for age group 2–5 years, but was not chosen for older age groups. Five out of six experts chose Emot2 as the best item for age groups 6–13 years and 14–17 years, though many were divided between Emot2 and Emot5. Parents chose Emot1 most often (4/6) for age group 2–5 years and chose Emot5 most often for age groups 6–13 years (3/6) and 14–17 years (2/3). The health status measurement expert reviewed all results and concluded that Emot5 may better express emotional functioning pathology compared to the other items since it is typical for children to experience some items in this dimension, such as Emot2 (“feeling sad or blue”). In fact, experiencing some level of such emotions may demonstrate better emotional functioning than if a child never experiences them. After careful consideration of all findings, the research team selected Emot5.

#### Social functioning

Items Soc2 (“others not wanting to be friends”) and Soc3 (“getting teased”) remained in this dimension after Step 1. Overall, Soc2 better fit the Rasch model, had larger item spread, less severe ceiling effects, and higher internal consistency compared to Soc3 (Appendix Tables 4D and 5C). None of the experts and parents thought that Soc3 was the best item to represent this dimension. In contrast, 2/6 experts chose Soc2 as the best item across all age groups and 3/6, 1/6, and 1/3 parents chose Soc2 as the best item for age groups 2–5 years, 6–13 years, and 14–17 years, respectively. Soc2 was selected for inclusion, and this decision was reviewed with the health status measurement expert who agreed that Soc2 was the most suitable item and best fit with the overall tone of the PedsUtil HSCS.

#### School functioning

School3 (“keeping up with schoolwork”) was the only remaining item after Step 1. To further validate item selection, the items were compared using Step 2 criteria. School3 better fit the Rasch model than the other two items, had the greatest item spread (Appendix Table 4E), and had high internal consistency (0.89) (Appendix Table 5D). However, School3 exhibited ceiling effects (≥ 26.5%). Nevertheless, all experts and parents agreed that School3 was the best item to represent this dimension and so this item was selected.

#### School absence

Only SchAbs1 (“missing school because sick”) remained after Step 1. SchAbs1 better fit the Rasch model and had higher correlation with the dimension score across age groups (0.90–0.91) (Appendix Tables 4F and 5E). Both SchAbs1 (≥ 38.3%) and SchAbs2 (≥ 56.3%) exhibited ceiling effects, although it was less severe for SchAbs1. All experts and parents agreed that SchAbs1 was the best item to represent this dimension, thus SchAbs1 was selected.

### Final PedsUtil health state classification system

**Fig. 2:**
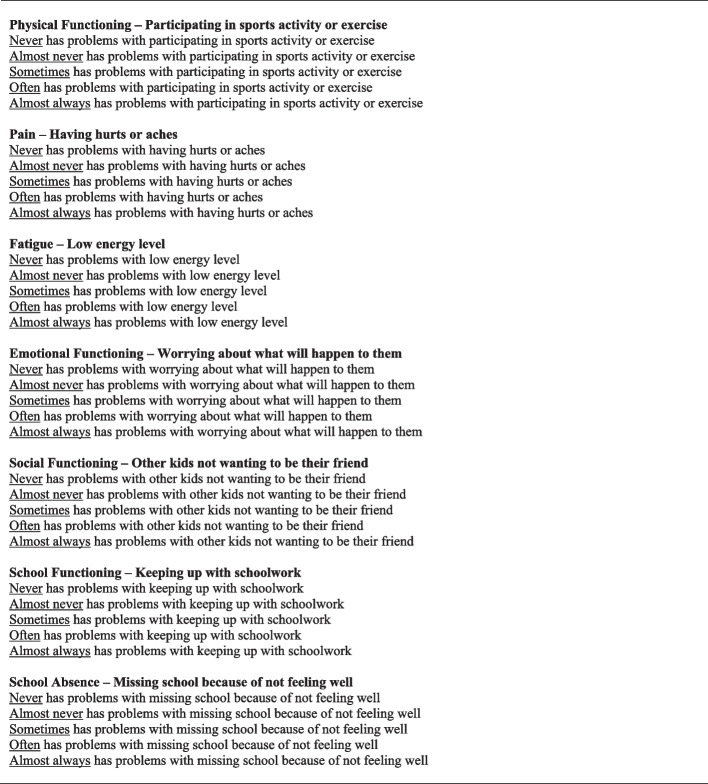
PedsUtil Health State Classification System^a^. ^a^Wording for PedsUtil health state classification system differs slightly between age groups but items selected are the same across all age groups. PedsUtil health state classification system for age group 8–12 years shown in this table

Figure 2 displays the final PedsUtil HSCS. Table 3 presents the Spearman’s correlation coefficients between the items selected to represent the dimensions across all ages. As shown in the table, there was minimal correlation, with most correlations ≤ 0.37. The only exception was for dimensions Pain and Fatigue, which had a correlation of 0.46. The limited correlations between the dimensions suggest that the dimensions are structurally independent.

**Table 3:** **Correlation Between Dimensions for All Ages**^a^

**Dimension**	**Physical Functioning** (“Participating in exercise”)	**Pain** (“Having hurts or aches”)	**Fatigue** (“Low energy level”)	**Emotional Functioning** (“Worrying”)	**Social Functioning** (“Others not wanting to be friends”)	**School Functioning** (“Keeping up with schoolwork”)	**School Absence** (“Missing school because sick”)
**Physical Functioning** (“Participating in exercise”)	1.00	–	–	–	–	–	–
**Pain** (“Having hurts or aches”)	0.24	1.00	–	–	–	–	–
**Fatigue** (“Low energy level”)	0.31	0.46	1.00	–	–	–	–
**Emotional Functioning** (“Worrying”)	0.20	0.32	0.34	1.00	–	–	–
**Social Functioning** (“Others not wanting to be friends”)	0.21	0.25	0.26	0.37	1.00	–	–
**School Functioning** (“Keeping up with schoolwork”)	0.35	0.19	0.25	0.26	0.28	1.00	–
**School Absence** (“Missing school because sick”)	0.23	0.33	0.32	0.25	0.19	0.28	1.00

^a^Age group specific correlations shown in Appendix Tables 7A-7C

## Discussion

Rasch analysis and various other psychometric assessments were utilized to derive the PedsUtil HSCS. Child health experts and parents were also involved in the item selection process to ensure content and face validity. The PedsUtil HSCS was constructed to be applicable to children 2–18 years. This is the first study to derive a HSCS based on the PedsQL.

The PedsQL has previously been mapped onto other preference-based utility measures, including the EQ-5D-Y [36] and CHU-9D [37–39]. Though these are alternative approaches to estimating health utilities from PedsQL responses, the mapping functions were estimated for very specific pediatric populations and for narrow age ranges. Therefore, current mapping functions are limited in their generalizability. The development of the PedsUtil scoring system, on the other hand, will allow for health utilities to be directly estimated from the PedsQL for children 2–18 years old.

When developing the PedsUtil HSCS, consideration was given to whether any wording or structure of the items needed to be changed to ensure that health states derived from the HSCS are amenable to valuation. For example, previous studies have explored collapsing item response levels because some respondents may find it difficult to distinguish between levels in preference valuation exercises [18, 21]. However, this study did not reduce the number of levels because doing so changes the original structure of the PedsQL, which may result in respondents valuing items with collapsed levels differently than if the original levels were preserved. Additionally, collapsing levels after selecting items may contradict Rasch criteria used earlier in the item selection process. Relatedly, other studies have also linked items to form a composite item to represent a single dimension [20, 22]. This study chose not to link items to best preserve the validity of PedsQL items that has previously been extensively researched. Moreover, respondents may value and interpret composite items differently compared to the original PedsQL items. The PedsQL is already widely used in clinical trials, research studies, and registries, thus maintaining its original wording helps ensure that data from these sources may be appropriately utilized for preference scoring. Furthermore, some design choices, such as combining item levels or altering dimension structure (e.g., deciding whether to retain both school absence and school functioning items), may be revisited and more appropriately addressed in the next phase of the study when such decisions can be informed by the performance of valuation models.

There are some limitations to this study and areas for further investigation. First, this study used parent-proxy responses to the PedsQL based on data availability. Future research should validate item selection with child self-report responses for age groups 5–7 years, 8–12 years, and 13–18 years. Second, data for the secondary analyses came from a general Australian population since the LSAC is one of the most extensive pediatric datasets with responses to the PedsQL. Though US-based experts and parents also aided in item selection, subsequent research is planned to validate the HSCS using data from other populations, including children with heterogeneous health conditions. Such analyses will also help address limitations of Rasch analysis encountered in this study where there was less variation in responses for some dimensions (e.g., Physical Functioning). Third, further work is needed to psychometrically test the PedsUtil HSCS, particularly the responsiveness of items to clinical change. PedsQL data from clinical trials would provide such insights. Fourth, the longitudinal design of the LSAC is potentially prone to limitations related to repeated measurement, such as order and learning effects. Fifth, the small and purposive sample of child health experts and parents used in this study may not reflect all viewpoints of the general population. Although participants were selected so that children of different ages and with different health conditions were widely represented, the resulting sample was mostly female and highly educated. Future research could further diversify the sample to investigate potential variations in opinions. Sixth, the age groups used in this analysis (i.e., 2–5 years, 6–13 years, and 14–17 years) were constructed to closely match those of the PedsQL (i.e., 2–4 years, 5–7 years, 8–12 years, and 13–18 years), though were not identical because of the study design of the LSAC. Specifically, the LSAC collects data every two years, requiring age groups to be grouped into two-year intervals. Children aged 6–7 years were also not constructed to be a separate age group, but instead were combined with children aged 8–13 years because children aged 6–7 years only represent a single wave of data collection. Prior work suggests that combining children aged 6–13 years may be appropriate as they represent the middle childhood years [40]. There was also some conflicting evidence across criteria for some items. For example, Emot2 (“feeling sad or blue”) performed poorly according to Rasch criteria, but many experts thought it was the best item for the Emotional Functioning dimension. No strict decision rules were applied to weight evidence across different criteria. Instead, final decisions were based on the research team’s collective judgment as done in previous studies [18–21]. Lastly, the PedsUtil HSCS may not be as sensitive as preference-based HRQoL instruments tailored specifically for narrow age ranges or developed de novo. However, given that the PedsQL is commonly used in clinical trials for pediatric interventions, developing the PedsUtil HSCS and its associated value sets facilitates the direct and consistent estimation of economic endpoints from the PedsQL without the need for additional resource-intensive data collection.

## Conclusion

This study identified the most representative item for each dimension to construct the PedsUtil HSCS. The items were selected based on Rasch analysis, psychometric methods, as well as input from child health experts and parents. Subsequent research will elicit preferences for the PedsUtil HSCS using valuation surveys to estimate a scoring system [41]. The PedsUtil scoring system will be one of the first preference-based HRQoL measures to estimate health utilities for children across a full range of ages 2–18 years, which will enable researchers to accurately and consistently value child health outcomes in health economic evaluations.

### Supplementary Information

Supplementary Material 1.

## Data Availability

The data that support the findings of this study are available from Australian Data Archive (ADA), but restrictions apply to the availability of these data, which were used under license for the current study and so are not publicly available. The data are, however, available from the authors upon reasonable request and with permission of Australian Data Archive.

## Abbreviations

HRQoL: Health-related quality of life
PedsQL: Pediatric Quality of Life Inventory
HSCS: Health state classification system
LSAC: Longitudinal Study of Australian Children
DIF: Differential item functioning
SchAbs: School Absence
School: School Functioning
Soc: Social Functioning
Emot: Emotional Functioning
Phys: Physical Functioning
