# Supplementary material for: Selecting PedsQL items to derive the PedsUtil health state classification system to measure health utilities in children
Source: Health Qual Life Outcomes. 2024 Jul 10;22:53. doi: 10.1186/s12955-024-02268-5 (PMC11238509; doi:10.1186/s12955-024-02268-5)
Supplement: Supplementary file 1 — Supplementary Material 1. [file 12955_2024_2268_MOESM1_ESM.docx]

**Table of Contents**

[Appendix Table 1. PedsQL Summary 3](#_Toc164688681)

[Appendix Table 2. Summary of LSAC Participants 4](#_Toc164688682)

[Appendix A. Rasch Criteria to Evaluate Item Exclusion 5](#_Toc164688683)

[Appendix B. Expert and Parent Opinion 7](#_Toc164688684)

[Appendix Table 3. Summary of Parent Participant Characteristics (*n* = 12) 8](#_Toc164688685)

[Appendix Figure 1. Sample Questions Provided to Experts and Parents 9](#_Toc164688686)

[Appendix Table 4A. Summary of Rasch Analysis Results – Physical Functioning 12](#_Toc164688687)

[Appendix Table 4B. Summary of Rasch Analysis Results – Physical Functioning Supplement 14](#_Toc164688688)

[Appendix Table 4C. Summary of Rasch Analysis Results – Emotional Functioning 15](#_Toc164688689)

[Appendix Table 4D. Summary of Rasch Analysis Results – Social Functioning 17](#_Toc164688690)

[Appendix Table 4E. Summary of Rasch Analysis Results – School Functioning 19](#_Toc164688691)

[Appendix Table 4F. Summary of Rasch Analysis Results – School Absence 20](#_Toc164688692)

[Appendix Table 5A. Summary of Psychometric Analysis and Expert and Parent Opinion – Physical Functioning 21](#_Toc164688693)

[Appendix Table 5B. Summary of Psychometric Analysis and Expert and Parent Opinion – Emotional Functioning 22](#_Toc164688694)

[Appendix Table 5C. Summary of Psychometric Analysis and Expert and Parent Opinion – Social Functioning 23](#_Toc164688695)

[Appendix Table 5D. Summary of Psychometric Analysis and Expert and Parent Opinion – School Functioning 24](#_Toc164688696)

[Appendix Table 5E. Summary of Psychometric Analysis and Expert and Parent Opinion – School Absence 25](#_Toc164688697)

[Appendix Table 6. Summary of Item Performance and Reason for Exclusion/Inclusion 26](#_Toc164688698)

[Appendix Table 7A. Correlation Between Dimensions for Age Group 2-5 Years 31](#_Toc164688699)

[Appendix Table 7B. Correlation Between Dimensions for Age Group 6-13 Years 32](#_Toc164688700)

[Appendix Table 7C. Correlation Between Dimensions for Age Group 14-17 Years 33](#_Toc164688701)

[References 34](#_Toc164688702)

# Appendix Table 1. PedsQL Summary

| **Dimensions** | **Number of Items** | | | |
| --- | --- | --- | --- | --- |
|  | **2-4 years**^a^ | **5-7 years**^a,b^ | **8-12 years**^a,b^ | **13-18 years**^a,b^ |
| Physical Functioning | 8 | 8 | 8 | 8 |
| Emotional Functioning | 5 | 5 | 5 | 5 |
| Social Functioning | 5 | 5 | 5 | 5 |
| School Functioning | 3 | 5 | 5 | 5 |
| **Total** | 21^c^ | 23 | 23 | 23 |

^a^ Parent proxy-report version available.

^b^ Child self-report version available.

^c^ PedsQL School Functioning contains 2 fewer items for children 2-4 years to reflect developmentally appropriate items.

# Appendix Table 2. Summary of LSAC Participants

| **Characteristic** | **2-5 Years** | | **6-13 Years** | | **14-17 Years** | |
| --- | --- | --- | --- | --- | --- | --- |
|  | **Full sample**  (*n* = 10,326) | **Rasch sample**^a^  (*n* = 3,506) | **Full sample**  (*n* = 28,558) | **Rasch sample**^a^  (*n* = 4,504) | **Full sample**  (*n* = 6,323) | **Rasch sample**^a^  (*n* = 4,004) |
| **CHILD** |  |  |  |  |  |  |
| Female [*n* (%)] | 5,049 (48.9) | 1,712 (48.8) | 13,917 (48.7) | 2,199 (48.8) | 3,117 (49.3) | 1,973 (49.3) |
| Child with Special Healthcare Needs  [*n* (%)] | 1,849 (17.9) | 618 (17.6) | 5,488 (19.2) | 869 (19.3) | 1,456 (23.0) | 924 (23.1) |
| PedsQL Total Score [mean (SD)] | 82.5 (9.9) | 82.3 (10.1) | 79.2 (13.1) | 79.0 (13.3) | 78.3 (14.7) | 78.4 (14.8) |
| Speak English at home | 9,467 (91.7) | 3,221 (91.9) | 26,171 (91.6) | 4,147 (92.1) | 5,755 (91.0) | 3,645 (91.0) |
| Number of Siblings |  |  |  |  |  |  |
| Single child | 1,353 (13.1) | 495 (14.1) | 2,433 (8.5) | 402 (8.9) | 880 (13.9) | 563 (14.1) |
| 1 sibling | 5,146 (49.8) | 1,770 (50.5) | 12,801 (44.8) | 1,953 (43.4) | 2,898 (45.8) | 1,827 (45.6) |
| ≥2 siblings | 3,827 (37.1) | 1,241 (35.4) | 13,324 (46.7) | 2,149 (47.7) | 2,545 (40.2) | 1,614 (40.3) |
| **PARENT**^b^ |  |  |  |  |  |  |
| Age, years  [mean (SD)] | 34.7 (5.3) | 34.4 (5.3) | 40.5 (5.7) | 40.4 (5.7) | 46.6 (5.3) | 46.5 (5.3) |
| Female [*n* (%)] | 10,099 (97.8) | 3,438 (98.1) | 27,481 (96.2) | 4,352 (96.6) | 5,917 (93.6) | 3,746 (93.6) |
| Education [*n* (%)] |  |  |  |  |  |  |
| Less than high  school | 116 (1.1) | 40 (1.1) | 323 (1.1) | 59 (1.3) | 65 (1.0) | 41 (1.0) |
| Some high school | 2,992 (29.0) | 959 (27.4) | 8,256 (28.9) | 1,285 (28.5) | 1,911 (30.2) | 1,244 (31.1) |
| High school graduate | 3,629 (35.1) | 1,245 (35.5) | 9,600 (33.6) | 1,518 (33.7) | 2,009 (31.8) | 1,269 (31.7) |
| College degree | 2,080 (20.1) | 723 (20.6) | 5,501 (19.3) | 843 (18.7) | 1,104 (17.5) | 681 (17.0) |
| Graduate degree | 1,505 (14.6) | 539 (15.4) | 4,847 (17.0) | 796 (17.7) | 1,223 (19.3) | 766 (19.1) |
| Two Parent Family, yes [*n* (%)] | 9,227 (89.4) | 3,145 (89.7) | 24,351 (85.3) | 3,840 (85.3) | 5,157 (81.6) | 3,252 (81.2) |
| Weekly Household Income, $1000 AU  [mean (SD)] | 1.8 (1.2) | 1.7 (1.2) | 2.2 (1.6) | 2.2 (1.6) | 2.7 (1.8) | 2.7 (1.8) |

Abbreviations: LSAC, Longitudinal Study of Australian Children; SD, standard deviation.

^a^ Subsamples that were used to conduct Rasch analyses (sampling stratified on child sex, age, and special healthcare needs status).

^b^ Primary parent that answered the PedsQL about their child.

# Appendix A. Rasch Criteria to Evaluate Item Exclusion

*Item Level Ordering*

The pattern of item response thresholds was first examined using individual item-threshold probability curves to determine if disordering was present. For a well-fitting item to the Rasch model, each item response level (i.e., Never, Almost Never, Sometimes, Often, and Almost Always) should systematically take turns showing the highest probability of being chosen. However, item misfit occurs when respondents inconsistently use the response levels. In other words, respondents are unable to distinguish between the item response levels. This may occur if there are too many item response levels or when the levels are poorly labeled or open to misinterpretation (1). Items that exhibit disordered thresholds fail to respond to the full range of severity across the dimension being measured, thus are not ideal to include in a health state classification system. For items that exhibited disordered thresholds, ordering of items was achieved by collapsing adjacent item response levels. Disordered items were assessed for exclusion from the PedsUtil health state classification system.

*Differential Item Functioning*

Once all items were ordered, the Rasch model was used to test for DIF. DIF can be a form of bias where responses to items systematically differ across respondent characteristics (e.g., males vs. females), despite having the same level of the underlying dimension being measured (2). For example, males and females with equal levels of physical ability may systematically respond differently to item(s) on the Physical Functioning dimension. However, any difference in scores observed for items exhibiting DIF may be biased because the difference may be an artifact of measurement nonequivalence rather than reflecting a true difference in scores between subgroups. Therefore, assessment of DIF yields crucial information about measurement invariance and may be be used to determine cross-population validity of items (2, 3).

Previous studies have documented gender differences in HRQoL outcomes (4, 5), thus this study examined DIF by sex in order to ensure that meaningful comparisons between males and females could be made using the PedsUtil health state classification system. Similarly, previous studies have tested for DIF by health status (6) thus this study examined DIF by child special healthcare needs status since the PedsUtil health state classification system needs to apply across diverse pediatric populations. It should be noted, however, that subjective judgment is often required to interpret whether DIF signifies bias (7). Therefore, DIF results were examined in conjunction with how the items performed on the other Rasch criteria to help determine overall item functioning. Two types of DIF were tested for in this study using analysis of variance – uniform and nonuniform DIF (8). Uniform DIF is when groups show a consistent difference in their responses to an item across the entire severity range of the dimension being measured (e.g., male responses are always higher than female responses). Nonuniform DIF is when the differences in responses between groups is not constant across the severity range (1, 2). For any items exhibiting DIF, the items were separated into different person factors and the Rasch model was refit. For example, if an item exhibited DIF by sex, the responses were separated for males and females. If splitting the item did not improve model fit, the item was considered for removal from the Rasch model.

Items that exhibit DIF may threaten construct validity and may be of limited value for making cross-population comparisons, thus were considered for exclusion from the PedsUtil health state classification system.

*Rasch Model Goodness-of-Fit*

After issues of disordered thresholds and DIF were resolved, Rasch model fit statistics were assessed to determine overall model goodness-of-fit. The overall model fit was specified by the item-trait interaction statistic, which was reported as a $\chi^{2}$ statistic. The item-trait interaction statistic reflects the property of invariance and a well-fitting Rasch model should exhibit the property of invariance. Accordingly, a statistically significant $\chi^{2}$ statistic indicates poor model fit. If overall model fit was statistically significant (i.e., *p*-value < 0.01 with a Bonferroni correction), the fit of the individual items was examined via fit residuals and individual item $\chi^{2}$ statistics. Fit residuals quantify the divergence between expected and observed responses and are standardized to approximate a *Z*-score, representing a standardized normal distribution. Items with fit residuals greater than the standard cutoff of $\pm$2.5 were considered to not fit the Rasch model (1, 6, 9). And similar to the overall model goodness-of-fit statistic, items with individual $\chi^{2}$ statistics that were statistically significant were also considered to not fit the Rasch model. In this analysis, poorly fitting items were dropped from the model sequentially, beginning with the worst fitting item. The Rasch model was refit after each item was removed. This process was repeated until only well-fitting items remained and the overall item-trait interaction statistic was nonsignificant. Items that were dropped from the Rasch model poorly represent the underlying dimension being measured, thus were considered for exclusion from the PedsUtil health state classification system.

# Appendix B. Expert and Parent Opinion

A convenience sample of US-based child health experts (pediatricians [*n* = 5] and a clinical trialist [*n* = 1]) were recruited to participate in expert panels, and US-based parents (*n* = 12) were recruited to participate in key informant interviews. Participants were asked to provide input on which item bests represent each dimension of the PedsUtil health state classification system. The experts were recruited through personal contacts of the research team, and parents were recruited through referrals from experts, online Facebook groups, and pediatrician offices affiliated with the University of Michigan Health System. All eligible participants were at least 18 years old and English speakers. Experts were compensated $75 and parents were compensated $25 for their participation.

A semi-structured interview format was used to conduct the expert panels and key informant interviews. All interviews were conducted online. Two main types of questions were asked. In cases where Rasch and psychometric analyses were able to identify a single item to represent a dimension, participants were asked if they agreed with the item selected. In cases where Rasch and psychometric analyses were only able to exclude some items but not identify a single best item, participants were asked to choose which item they believed best represents the dimension among the remaining items and to provide justifications for their choices. Participants could also disagree with the item(s) excluded based on Rasch and psychometric criteria and could instead select a different item from the full list of items in each dimension as the best item to represent that dimension. Experts were asked to provide input for all age groups, whereas parents were asked to provide feedback for age groups that correspond to their children’s current age. **Appendix Figure 1** provides sample questions regarding item selection that were presented to experts and parents.

# Appendix Table 3. Summary of Parent Participant Characteristics (*n* = 12)

| Characteristic | *n* | % |
| --- | --- | --- |
| Gender |  |  |
| Male | 1 | 8% |
| Female | 11 | 92% |
| Highest level of education |  |  |
| High school/GED | 0 | 0% |
| 2-year college/Associate’s degree | 0 | 0% |
| 4-year college degree | 1 | 8% |
| Advanced degree (Master’s,  Doctorate or Professional) | 7 | 58% |
| Unknown | 4 | 33% |
| Number of children |  |  |
| 1 | 3 | 25% |
| 2 | 8 | 67% |
| 3 or more | 1 | 8% |
| Age of child(ren)^a^ |  |  |
| 2-5 years | 6 | –^b^ |
| 6-13 years | 6 | – |
| 14-17 years | 3 | – |
| Child health status^c^ |  |  |
| Typically functioning child | 8 | –^b^ |
| Child with special healthcare needs | 8 | – |

^a^ Some parents had children in different age groups so total adds up to more than *n* = 12.

^b^ % not calculated because total equals to more than *n* = 12 since parents can be in multiple categories.

^c^ Some parents had both typically functioning children and children with special healthcare needs so total adds up to more than *n* = 12.

# Appendix Figure 1. Sample Questions Provided to Experts and Parents

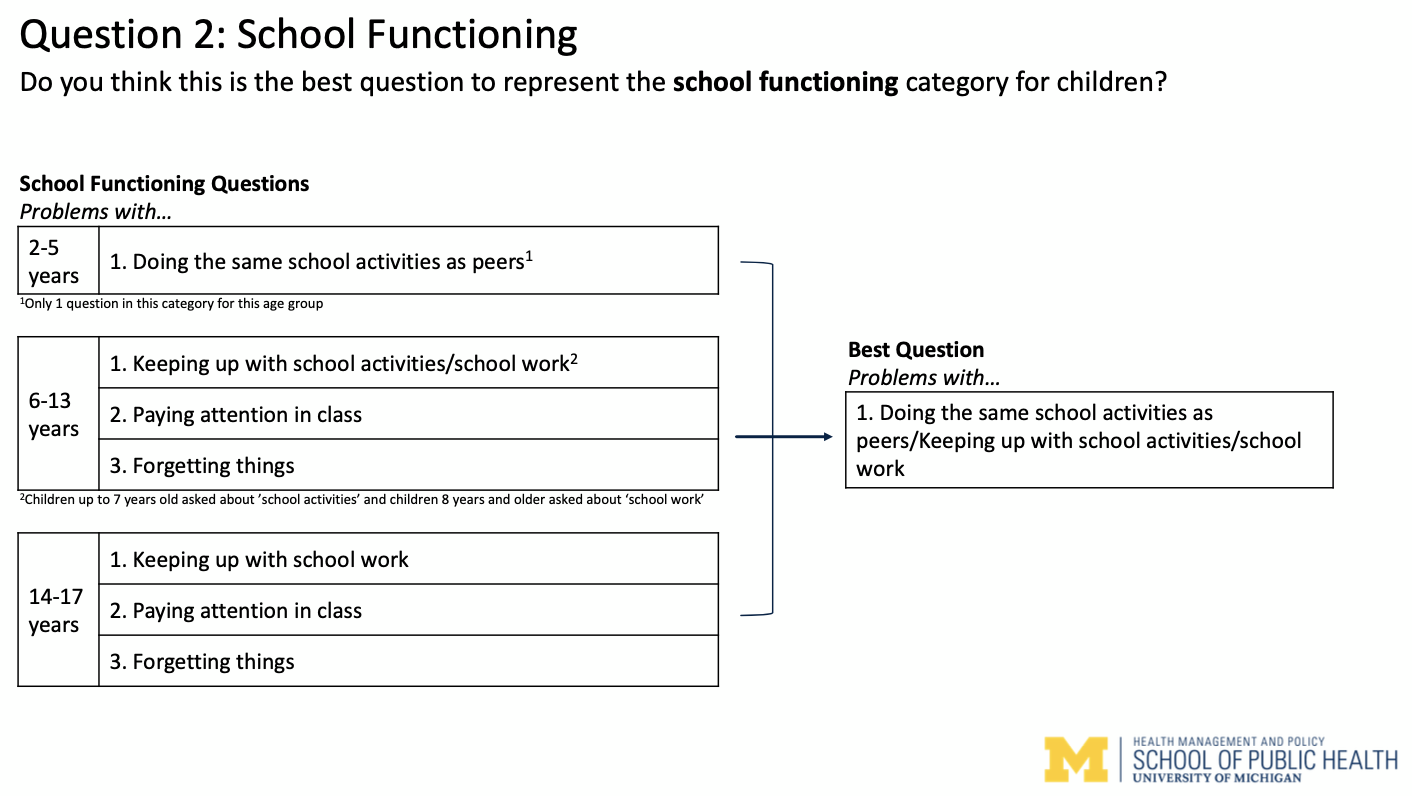


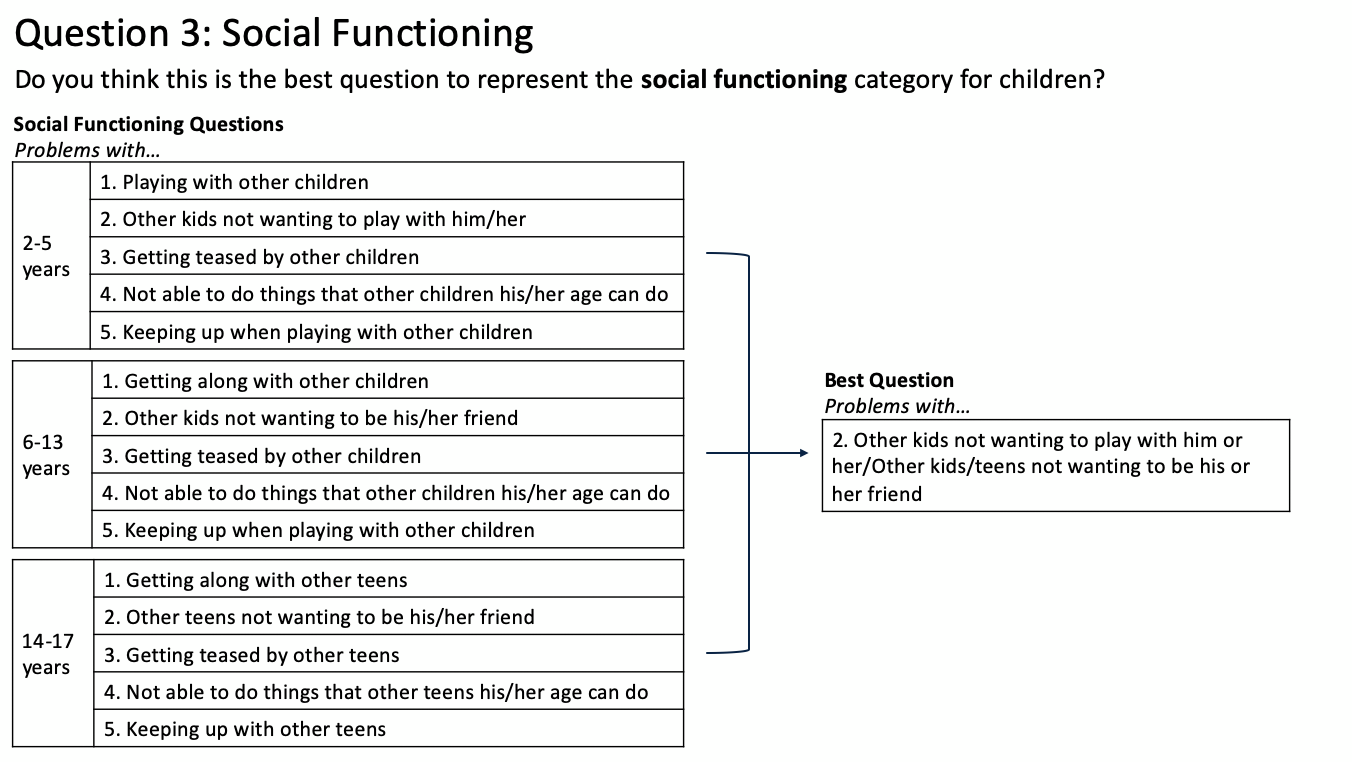


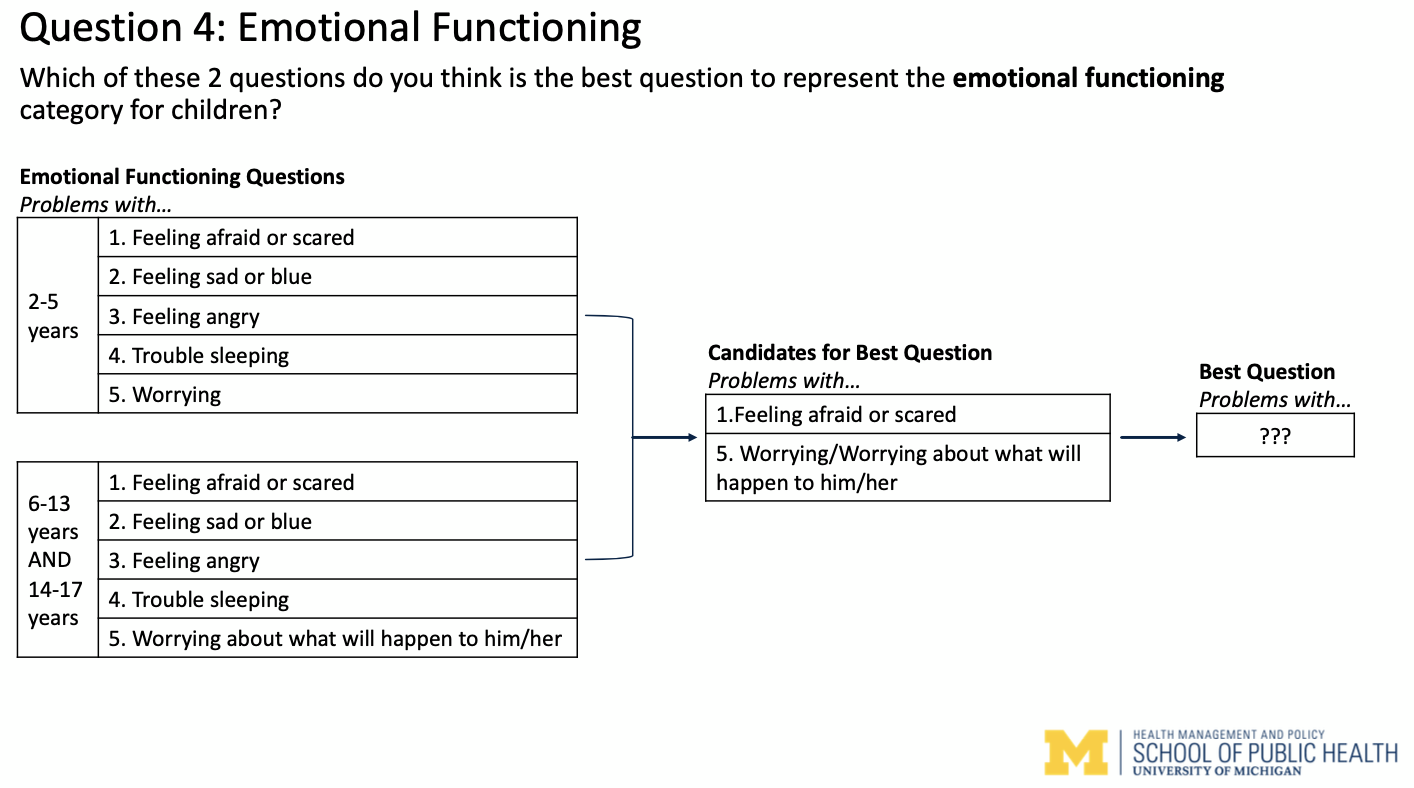


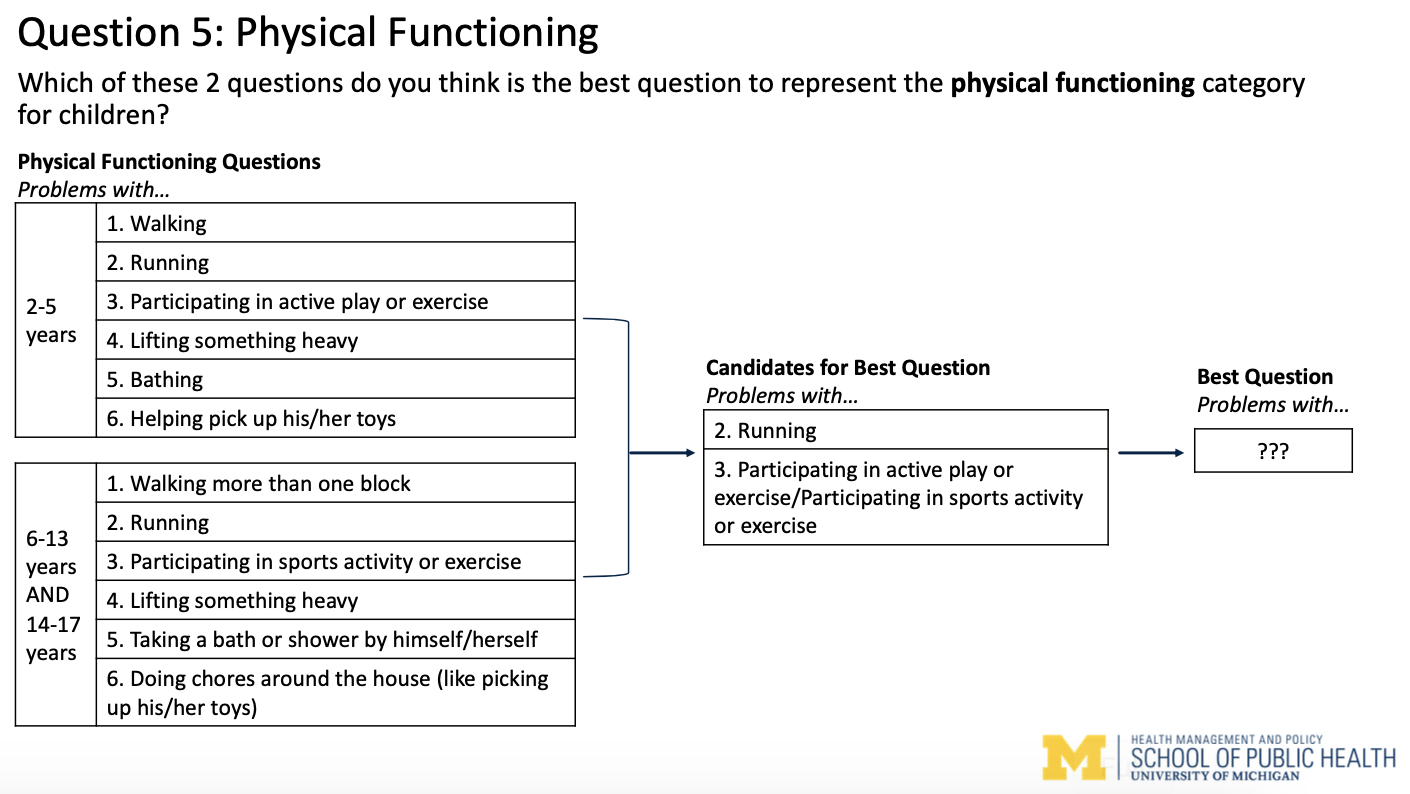


# Appendix Table 4A. Summary of Rasch Analysis Results – Physical Functioning

| **Item Description**  *Problems with…* | **Total Score**^a^ | **Mean Item Level Performance**^b^  (Range Across Subsamples) | | | **Disordered Thresholds** | **DIF**^c^ | **Item Misfit** |
| --- | --- | --- | --- | --- | --- | --- | --- |
|  |  | ***p*-value**^d^ | **Fit Residual**^e^ | **Spread** |  |  |  |
| **2-5 years**^f^ | | | | | | | |
| Phys1. Walking | 0/5 | – | – | – | 5 samples | 1 sample  (SHCN status^g^) | 4 samples |
| Phys2. Running | 0/5 | – | – | – | 5 samples | No DIF | 3 samples |
| Phys3. Participating in  exercise | 1/5 | 0.01^h^ | 2.12 | 0.46 | 4 samples | No DIF | 3 samples |
| Phys4. Lifting something  heavy | 0/5 | – | – | – | 4 samples | 4 samples (Sex) | No misfit |
| Phys5. Taking a bath or  shower | 0/5 | – | – | – | 5 samples | No DIF | 4 samples |
| Phys6. Doing chores | 0/5 | – | – | – | 2 samples | No DIF | 4 samples |
| **6-13 years**^e^ | | | | | | | |
| Phys1. Walking | 0/5 | – | – | – | 5 samples | No DIF | 4 samples |
| Phys2. Running | 0/5 | – | – | – | 2 samples | 1 sample (Sex) | 4 samples |
| Phys3. Participating in  exercise | 1/5 | 0.22^h^ | 0.17 | 0.67 | 1 sample | No DIF | 4 samples |
| Phys4. Lifting something  heavy | 0/5 | – | – | – | 5 samples | No DIF | 5 samples |
| Phys5. Taking a bath or  shower | 0/5 | – | – | – | 5 samples | 2 samples  (SHCN status) | 2 samples |
| Phys6. Doing chores | 0/5 | – | – | – | 1 sample | 1 sample (Sex)  1 sample  (SHCN status) | 5 samples |
| **14-17 years** | | | | | | | |
| Phys1. Walking | 0/5 | – | – | – | 5 samples | 1 sample (Sex) | 3 samples |
| Phys2. Running | 3/5 | 0.36  (0.05, 0.58) | 0.61  (0.26, 1.11) | 0.56  (0.49, 0.67) | None | 1 sample (Sex)  1 sample  (SHCN status) | No misfit |
| Phys3. Participating in  exercise | 2/5 | 0.13  (0.003, 0.25) | 2.04  (1.74, 2.35) | 0.65  (0.60, 0.70) | None | 1 sample (Sex)  2 samples  (SHCN status) | 1 sample |
| Phys4. Lifting something  heavy | 3/5 | 0.51  (0.34, 0.63) | 1.34  (0.50, 1.93) | 0.64  (0.62, 0.65) | 1 sample | No DIF | 2 samples |
| Phys5. Taking a bath or  shower | 0/5 | – | – | – | 5 samples | 1 sample (Sex)  1 sample  (SHCN status)  1 sample  (SHCN status  and Sex) | No misfit |
| Phys6. Doing chores | 0/5 | – | – | – | None | 4 samples (Sex)  1 sample  (SHCN status) | 5 samples |

Abbreviations: DIF, differential item functioning; Phys, Physical Functioning; SHCN, special healthcare needs.

^a^ Total score = number of subsamples item performed well on all Rasch criteria (out of five subsamples).

^b^ Mean item level performance calculated using only the subsamples that the item performed well on all Rasch criteria.

^c^ A *p*-value < 0.05 (with Bonferroni adjustment) was used to determine presence of DIF.

^d^ *p*-value for the individual item $\chi^{2}$ statistics.

^e^ Fit residuals may be positive or negative, thus absolute value of the fit residuals reported in the table.

^f^ 4 subsamples did not fit the Rasch model.

^g^ Special healthcare needs status defined as children with special healthcare needs or typically functioning children.

^h^ Only one subsample performed well on all Rasch criteria, thus no range reported in table.

# Appendix Table 4B. Summary of Rasch Analysis Results – Physical Functioning Supplement^a^

| **Item Description**  *Problems with…* | **Total Score**^b^ | **Mean Item Level Performance**^c^  (Range Across Subsamples) | | | **Disordered Thresholds** | **DIF**^d^ | **Item Misfit** |
| --- | --- | --- | --- | --- | --- | --- | --- |
|  |  | ***p*-value**^e^ | **Fit Residual**^f^ | **Spread** |  |  |  |
| **2-5 years**^g^ | | | | | | | |
| Phys1. Walking | 0/3^h^ | – | – | – | 1 sample | No DIF | 3 samples |
| Phys2. Running | 0/3 | – | – | – | 2 samples | No DIF | 3 samples |
| Phys3. Participating in  exercise | 0/3 | – | – | – | 3 samples | No DIF | 3 samples |
| **6-13 years**^i^ | | | | | | | |
| Phys1. Walking | 1/5 | 0.02^j^ | 0.94 | 0.47 | None | No DIF | 3 samples |
| Phys2. Running | 4/5 | 0.08  (0.004, 0.13) | 1.13  (1.09, 1.17) | 0.68  (0.64, 0.72) | None | No DIF | 1 sample |
| Phys3. Participating in  exercise | 4/5 | 0.26  (0.06, 0.63) | 0.42  (0.08, 1.16) | 0.59  (0.54, 0.64) | 1 sample | No DIF | 1 sample |
| **14-17 years** | | | | | | | |
| Phys1. Walking | 1/3^h^ | 0.01^j^ | 0.20 | 0.22 | 1 sample | No DIF | 2 samples |
| Phys2. Running | 3/3 | 0.06  (0.02, 0.09) | 0.70  (0.11, 1.10) | 0.63  (0.56, 0.78) | None | No DIF | None |
| Phys3. Participating in  exercise | 3/3 | 0.05  (0.02, 0.13) | 0.37  (0.002, 0.57) | 0.61  (0.50, 0.72) | None | No DIF | None |

Abbreviations: DIF, differential item functioning; Phys, Physical Functioning.

^a^ Supplemental analyses excluded items Phy4-Phys6 as they were considered not as relevant for this dimension.

^b^ Total score = number of subsamples item performed well on all Rasch criteria (out of five subsamples).

^c^ Mean item level performance calculated using only the subsamples that the item performed well on all Rasch criteria.

^d^ A *p*-value < 0.05 (with Bonferroni adjustment) was used to determine presence of DIF.

^e^ *p*-value for the individual item $\chi^{2}$ statistics.

^f^ Fit residuals may be positive or negative, thus absolute value of the fit residuals reported in the table.

^g^ None of the subsamples fit the Rasch model.

^h^ Insufficient sample size to obtain five subsamples so only three subsamples were created for supplemental analyses (so total score out of 3).

^i^ One out of the five subsamples did not fit the overall Rasch model.

^j^ Only one subsample performed well on all Rasch criteria, thus no range reported in table.

# Appendix Table 4C. Summary of Rasch Analysis Results – Emotional Functioning

| **Item Description**  *Problems with…* | **Total Score**^a^ | **Mean Item Level Performance**^b^  (Range Across Subsamples) | | | **Disordered Thresholds** | **DIF**^c^ | **Item Misfit** |
| --- | --- | --- | --- | --- | --- | --- | --- |
|  |  | ***p*-value**^d^ | **Fit Residual**^e^ | **Spread** |  |  |  |
| **2-5 years** | | | | | | | |
| Emot1. Feeling afraid or  scared | 4/5 | 0.16  (0.05, 0.40) | 0.90  (0.30, 1.38) | 1.10  (0.99, 1.28) | 1 sample | No DIF | No misfit |
| Emot2. Feeling sad or blue | 1/5 | 0.36^f^ | 0.50 | 1.11 | 4 samples | No DIF | No misfit |
| Emot3. Feeling angry | 4/5 | 0.51  (0.09, 0.84) | 0.72  (0.26, 1.72) | 1.57  (1.24, 1.97) | 1 sample | 1 sample (Sex) | No misfit |
| Emot4. Trouble sleeping | 2/5 | 0.36  (0.34, 0.37) | 0.37  (0.25, 0.49) | 0.47  (0.43, 0.50) | 1 sample | No DIF | 2 samples |
| Emot5. Worrying | 4/5 | 0.05  (0.004, 0.11) | 1.26  (0.03, 1.81) | 0.99  (0.76, 1.28) | None | No DIF | 1 sample |
| **6-13 years** | | | | | | | |
| Emot1. Feeling afraid or  scared | 2/5 | 0.27  (0.08, 0.45) | 1.13  (0.86, 1.41) | 1.09  (0.96, 1.22) | 1 sample | 2 samples (Sex) | No misfit |
| Emot2. Feeling sad or blue | 5/5 | 0.13  (0.007, 0.43) | 0.71  (0.25, 1.44) | 1.20  (0.89, 1.58) | None | No DIF | No misfit |
| Emot3. Feeling angry | 1/5 | 0.25^f^ | 1.13 | 0.97 | None | 3 samples (Sex)  1 sample  (SHCN status^g^  and Sex) | 4 samples |
| Emot4. Trouble sleeping | 0/5 | – | – | – | 2 samples | No DIF | 4 samples |
| Emot5. Worrying | 5/5 | 0.25  (0.04, 0.51) | 0.52  (0.38, 0.74) | 0.98  (0.75, 1.32) | None | No DIF | No misfit |
| **14-17 years** | | | | | | | |
| Emot1. Feeling afraid or  scared | 2/5 | 0.06  (0.005, 0.12) | 1.23  (0.85, 1.62) | 0.94  (0.76, 1.12) | 1 sample | 2 samples (Sex)  1 sample  (SHCN status  and Sex) | No misfit |
| Emot2. Feeling sad or blue | 0/5 | – | – | – | None | 4 samples (Sex)  1 sample  (SHCN status  and Sex) | 2 samples |
| Emot3. Feeling angry | 1/5 | 0.57^f^ | 1.50 | 1.08 | None | 3 samples (Sex) | 1 sample |
| Emot4. Trouble sleeping | 2/5 | 0.30  (0.17, 0.43) | 2.09  (1.92, 2.26) | 0.62  (0.57, 0.67) | None | No DIF | 3 samples |
| Emot5. Worrying | 5/5 | 0.33  (0.01, 0.68) | 1.20  (0.47, 1.69) | 0.99  (0.81, 1.12) | None | No DIF | No misfit |

Abbreviations: DIF, differential item functioning; Emot, Emotional Functioning; SHCN, special healthcare needs.

^a^ Total score = number of subsamples item performed well on all Rasch criteria (out of five subsamples).

^b^ Mean item level performance calculated using only the subsamples that the item performed well on all Rasch criteria.

^c^ A *p*-value < 0.05 (with Bonferroni adjustment) was used to determine presence of DIF.

^d^ *p*-value for the individual item $\chi^{2}$ statistics.

^e^ Fit residuals may be positive or negative, thus absolute value of the fit residuals reported in the table.

^f^ Only one subsample performed well on all Rasch criteria, thus no range reported in table.

^g^ Special healthcare needs status defined as children with special healthcare needs or typically functioning children.

# Appendix Table 4D. Summary of Rasch Analysis Results – Social Functioning

| **Item Description**  *Problems with…* | **Total Score**^a^ | **Mean Item Level Performance**^b^  (Range Across Subsamples) | | | **Disordered Thresholds** | **DIF**^c^ | **Item Misfit** |
| --- | --- | --- | --- | --- | --- | --- | --- |
|  |  | ***p*-value**^d^ | **Fit Residual**^e^ | **Spread** |  |  |  |
| **2-5 years** | | | | | | | |
| Soc1. Getting along with  others | 0/5 | – | – | – | 5 samples | No DIF | No misfit |
| Soc2. Others not wanting  to be friends | 4/5 | 0.11  (0.01, 0.36) | 1.28  (0.18, 2.23) | 1.14  (0.88, 1.34) | 1 sample | No DIF | No misfit |
| Soc3. Getting teased | 4/5 | 0.10  (0.01, 0.27) | 1.05  (0.63, 2.15) | 1.02  (0.81, 1.18) | 1 sample | No DIF | No misfit |
| Soc4. Unable to do things  others can do | 1/5 | 0.14^f^ | 0.84 | 0.52 | 4 samples | 1 sample  (SHCN status^g^)  1 sample (Sex) | No misfit |
| Soc5. Keeping up with  other children | 0/5 | – | – | – | 5 samples | No DIF | No misfit |
| **6-13 years** | | | | | | | |
| Soc1. Getting along with  others | 0/5 | – | – | – | 5 samples | 1 sample  (SHCN status)  1 sample (Sex) | 1 sample |
| Soc2. Others not wanting  to be friends | 4/5 | 0.24  (0.02, 0.90) | 1.17  (0.42, 1.64) | 1.25  (0.75, 1.84) | 1 sample | 1 sample (Sex) | No misfit |
| Soc3. Getting teased | 1/5 | 0.22^f^ | 0.31 | 0.92 | 3 samples | 1 sample  (SHCN status) | No misfit |
| Soc4. Unable to do things  others can do | 0/5 | – | – | – | 5 samples | No DIF | 2 samples |
| Soc5. Keeping up with  other children | 0/5 | – | – | – | 5 samples | 1 sample  (SHCN status) | 2 samples |
| **14-17 years** | | | | | | | |
| Soc1. Getting along with  others | 0/5 | – | – | – | 5 samples | 1 sample  (SHCN status) | No misfit |
| Soc2. Others not wanting  to be friends | 3/5 | 0.17  (0.09, 0.27) | 0.65  (0.46, 1.0) | 1.04  (0.76, 1.35) | 1 sample | 2 samples (Sex) | No misfit |
| Soc3. Getting teased | 2/5 | 0.57  (0.44, 0.70) | 0.43  (0.01, 0.86) | 0.87  (0.77, 0.97) | 2 samples | 1 sample (Sex) | No misfit |
| Soc4. Unable to do things  others can do | 0/5 | – | – | – | 4 samples | 1 sample  (SHCN status)  1 sample (Sex) | No misfit |
| Soc5. Keeping up with  other children | 0/5 | – | – | – | 5 samples | No DIF | No misfit |

Abbreviations: DIF, differential item functioning; SHCN, special healthcare needs; Soc, Social Functioning.

^a^ Total score = number of subsamples item performed well on all Rasch criteria (out of five subsamples).

^b^ Mean item level performance calculated using only the subsamples that the item performed well on all Rasch criteria.

^c^ A *p*-value < 0.05 (with Bonferroni adjustment) was used to determine presence of DIF.

^d^ *p*-value for the individual item $\chi^{2}$ statistics.

^e^ Fit residuals may be positive or negative, thus absolute value of the fit residuals reported in the table.

^f^ Only one subsample performed well on all Rasch criteria, thus no range reported in table.

^g^ Special healthcare needs status defined as children with special healthcare needs or typically functioning children.

# Appendix Table 4E. Summary of Rasch Analysis Results – School Functioning

| **Item Description**  *Problems with…* | **Total Score**^a^ | **Mean Item Level Performance**^b^  (Range Across Subsamples) | | | **Disordered Thresholds** | **DIF**^c^ | **Item Misfit** |
| --- | --- | --- | --- | --- | --- | --- | --- |
|  |  | ***p*-value**^d^ | **Fit Residual**^e^ | **Spread** |  |  |  |
| **2-5 years** | | | | | | | |
| School3. Keeping up with  schoolwork | Only item included – Rasch analysis not performed^f^ | | | | | | |
| **6-13 years** | | | | | | | |
| School1. Paying attention  in class | 0/5 | – | – | – | 2 samples | 5 samples (Sex) | No misfit |
| School2. Forgetting things | 0/5 | – | – | – | None | No DIF | 5 samples |
| School3. Keeping up with  schoolwork | 0/5 | – | – | – | 3 samples | 5 samples (Sex) | No misfit |
| **14-17 years** | | | | | | | |
| School1. Paying attention  in class | 2/5 | 0.08  (0.06, 0.10) | 0.45  (0.43, 0.47) | 1.10  (1.06, 1.15) | None | 3 samples (Sex) | No misfit |
| School2. Forgetting things | 0/5 | – | – | – | None | 1 sample  (SHCN status^g^  and Sex) | 5 samples |
| School3. Keeping up with  schoolwork | 4/5 | 0.83  (0.76, 0.87) | 0.44  (0.34, 0.60) | 1.13  (1.08, 1.22) | 1 sample | 1 sample (Sex) | No misfit |

Abbreviations: DIF, differential item functioning; SHCN, special healthcare needs.

^a^ Total score = number of subsamples item performed well on all Rasch criteria (out of five subsamples).

^b^ Mean item level performance calculated using only the subsamples that the item performed well on all Rasch criteria.

^c^ A *p*-value < 0.05 (with Bonferroni adjustment) was used to determine presence of DIF.

^d^ *p*-value for the individual item $\chi^{2}$ statistics.

^e^ Fit residuals may be positive or negative, thus absolute value of the fit residuals reported in the table.

^f^ Only School3 is included in the PedsQL for this dimension for this age group, thus Rasch analysis was not performed.

^g^ Special healthcare needs status defined as children with special healthcare needs or typically functioning children.

# Appendix Table 4F. Summary of Rasch Analysis Results – School Absence^a^

| **Item Description**  *Problems with…* | **Total Score**^b^ | **Mean Item Level Performance**^c^  (Range Across Subsamples) | | | **Disordered Thresholds** | **DIF**^d^ | **Item Misfit** |
| --- | --- | --- | --- | --- | --- | --- | --- |
|  |  | ***p*-value**^e^ | **Fit Residual**^f^ | **Spread** |  |  |  |
| **2-5 years**^g^ | | | | | | | |
| SchAbs1. Missing school  because sick | 2/5 | 0.15  (0.10, 0.19) | 0.37  (0.17, 0.57) | 2.08  (1.86, 2.30) | 1 sample | 2 samples  (SHCN status^h^) | No misfit |
| SchAbs2. Missing school  to go to doctor | 0/5 | – | – | – | 1 sample | 2 samples  (SHCN status) | 5 samples |
| **6-13 years** | | | | | | | |
| SchAbs1. Missing school  because sick | 4/5 | 0.06  (0.01, 0.15) | 0.53  (0.33, 0.73) | 1.67  (1.10, 2.03) | None | No DIF | 1 sample |
| SchAbs2. Missing school  to go to doctor | 0/5 | – | – | – | 1 sample | 2 samples  (SHCN status) | 5 samples |
| **14-17 years** | | | | | | | |
| SchAbs1. Missing school  because sick | 3/5 | 0.04  (0.02, 0.06) | 0.09  (0.02, 0.16) | 1.22  (1.14, 1.32) | None | No DIF | 2 samples |
| SchAbs2. Missing school  to go to doctor | 0/5 | – | – | – | None | 1 sample  (SHCN status) | 5 samples |

Abbreviations: DIF, differential item functioning; SchAbs, School Absence; SHCN, special healthcare needs.

^a^ All subsamples across all age groups did not fit the overall Rasch model, thus the item-specific results reported in the table are for Rasch models with statistically significant item-trait interaction $\chi^{2}$ statistics.

^b^ Total score = number of subsamples item performed well on all Rasch criteria (out of five subsamples).

^c^ Mean item level performance calculated using only the subsamples that the item performed well on all Rasch criteria.

^d^ A *p*-value < 0.05 (with Bonferroni adjustment) was used to determine presence of DIF.

^e^ *p*-value for the individual item $\chi^{2}$ statistics.

^f^ Fit residuals may be positive or negative, thus absolute value of the fit residuals reported in the table.

^g^ School Absence dimension was not asked for children aged 2-3 years in the LSAC, thus results reflect responses for children aged 4-5 years.

^h^ Special healthcare needs status defined as children with special healthcare needs or typically functioning children.

# Appendix Table 5A. Summary of Psychometric Analysis and Expert and Parent Opinion – Physical Functioning

| **Item Description**  *Problems with…* | **Psychometric Analysis** | | | **Expert and Parent Opinion** | |
| --- | --- | --- | --- | --- | --- |
|  | **% Response Ceiling (Never)** | **% Response Floor**  **(Almost always)** | **Internal Consistency**^a^ | **Best item – Experts**^b^ | **Best item – Parents**^c^ |
| **2-5 years** | | | |  |  |
| Phys1. Walking | 93.1% | 0.2% | 0.58^d^ | 0/6 | 0/6 |
| Phys2. Running | 91.8% | 0.3% | 0.62^e^ | 0/6 | 1/6 |
| Phys3. Participating in  exercise | 80.5% | 0.6% | 0.67^f^ | 5/6 | 5/6 |
| Phys4. Lifting something  heavy | 54.6% | 0.5% | 0.64 | 0/6 | 0/6 |
| Phys5. Taking a bath or  shower | 77.5% | 0.8% | 0.64 | 1/6 | 0/6 |
| Phys6. Doing chores | 22.1% | 3.8% | 0.64 | 0/6 | 0/6 |
| **6-13 years** | | | |  |  |
| Phys1. Walking | 83.1% | 1.5% | 0.81^d^ | 0/6 | 0/6 |
| Phys2. Running | 76.0% | 1.8% | 0.82^e^ | 0/6 | 0/6 |
| Phys3. Participating in  exercise | 73.1% | 3.1% | 0.86^f^ | 5/6 | 6/6 |
| Phys4. Lifting something  heavy | 63.2% | 1.0% | 0.71 | 0/6 | 0/6 |
| Phys5. Taking a bath or  shower | 78.9% | 5.1% | 0.78 | 1/6 | 0/6 |
| Phys6. Doing chores | 36.5% | 4.3% | 0.65 | 0/6 | 0/6 |
| **14-17 years** | | | |  |  |
| Phys1. Walking | 82.3% | 2.5% | 0.84^d^ | 0/6 | 0/3 |
| Phys2. Running | 70.6% | 2.3% | 0.82^e^ | 0/6 | 0/3 |
| Phys3. Participating in  exercise | 67.7% | 3.5% | 0.87^f^ | 5/6 | 3/3 |
| Phys4. Lifting something  heavy | 69.1% | 1.5% | 0.80 | 0/6 | 0/3 |
| Phys5. Taking a bath or  shower | 87.5% | 8.2% | 0.79 | 1/6 | 0/3 |
| Phys6. Doing chores | 46.7% | 3.9% | 0.67 | 0/6 | 0/3 |

Abbreviations: Phys, Physical Functioning.

^a^ Correlation of item score with dimension score (i.e., Spearman’s correlation coefficient).

^b^ Number of experts (out of *n* = 6) that chose an item as the best item to represent this dimension.

^c^ Number of parents that chose an item as the best item to represent this dimension. There were *n* = 6 parents with children 2-5 years old, *n* = 6 parents with children 6-13 years old, and *n* = 3 parents for children 14-17 years old (*n* = 12 parents but some parents had children in different age groups so total adds up to more than 12).

^d^ Correlation of Phys 1 with Physical Functioning dimension if the dimension included only three items (Phys1-Phys3) was 0.81 for age group 2-5 years, 0.88 for age group 6-13 years, and 0.86 for age group 14-17 years.

^e^ Correlation of Phys 2 with Physical Functioning dimension if the dimension included only three items (Phys1-Phys3) was 0.86 for age group 2-5 years, 0.93 for age group 6-13 years, and 0.92 for age group 14-17 years.

^f^ Correlation of Phys 3 with Physical Functioning dimension if the dimension included only three items (Phys1-Phys3) was 0.83 for age group 2-5 years, 0.91 for age group 6-13 years, and 0.91 for age group 14-17 years.

# Appendix Table 5B. Summary of Psychometric Analysis and Expert and Parent Opinion – Emotional Functioning

| **Item Description**  *Problems with…* | **Psychometric Analysis** | | | **Expert and Parent Opinion** | |
| --- | --- | --- | --- | --- | --- |
|  | **% Response Ceiling (Never)** | **% Response Floor**  **(Almost always)** | **Internal Consistency**^a^ | **Best item – Experts**^b^ | **Best item – Parents**^c^ |
| **2-5 years** | | | |  |  |
| Emot1. Feeling afraid or  scared | 23.0% | 0.3% | 0.72 | 3/6 | 4/6 |
| Emot2. Feeling sad or blue | 31.0% | 0.1% | 0.71 | 2/6 | 0/6 |
| Emot3. Feeling angry | 12.2% | 0.2% | 0.67 | 0/6 | 0/6 |
| Emot4. Trouble sleeping | 36.7% | 1.9% | 0.65 | 0/6 | 0/6 |
| Emot5. Worrying | 50.9% | 0.2% | 0.72 | 1/6 | 2/6 |
| **6-13 years** | | | |  |  |
| Emot1. Feeling afraid or  scared | 35.8% | 0.5% | 0.77 | 0/6 | 1/6 |
| Emot2. Feeling sad or blue | 31.2% | 0.3% | 0.77 | 5/6 | 2/6 |
| Emot3. Feeling angry | 15.4% | 0.7% | 0.69 | 0/6 | 0/6 |
| Emot4. Trouble sleeping | 42.3% | 1.9% | 0.71 | 0/6 | 0/6 |
| Emot5. Worrying | 39.7% | 0.9% | 0.77 | 1/6 | 3/6 |
| **14-17 years** | | | |  |  |
| Emot1. Feeling afraid or  scared | 53.0% | 0.5% | 0.78 | 0/6 | 0/3 |
| Emot2. Feeling sad or blue | 31.4% | 0.7% | 0.84 | 5/6 | 1/3 |
| Emot3. Feeling angry | 19.0% | 1.0% | 0.75 | 0/6 | 0/3 |
| Emot4. Trouble sleeping | 34.5% | 3.1% | 0.77 | 0/6 | 0/3 |
| Emot5. Worrying | 36.6% | 1.1% | 0.81 | 1/6 | 2/3 |

Abbreviations: Emot, Emotional Functioning.

^a^ Correlation of item score with dimension score (i.e., Spearman’s correlation coefficient).

^b^ Number of experts (out of *n* = 6) that chose an item as the best item to represent this dimension.

^c^ Number of parents that chose an item as the best item to represent this dimension. There were *n* = 6 parents with children 2-5 years old, *n* = 6 parents with children 6-13 years old, and *n* = 3 parents for children 14-17 years old (*n* = 12 parents but some parents had children in different age groups so total adds up to more than 12).

# Appendix Table 5C. Summary of Psychometric Analysis and Expert and Parent Opinion – Social Functioning

| **Item Description**  *Problems with…* | **Psychometric Analysis** | | | **Expert and Parent Opinion** | |
| --- | --- | --- | --- | --- | --- |
|  | **% Response Ceiling (Never)** | **% Response Floor**  **(Almost always)** | **Internal Consistency**^a^ | **Best item – Experts**^b^ | **Best item – Parents**^c^ |
| **2-5 years** | | | |  |  |
| Soc1. Getting along with  others | 48.8% | 1.6% | 0.74 | 4/6 | 3/6 |
| Soc2. Others not wanting  to be friends | 46.8% | 0.2% | 0.75 | 2/6 | 3/6 |
| Soc3. Getting teased | 60.8% | 0.1% | 0.68 | 0/6 | 0/6 |
| Soc4. Unable to do things  others can do | 63.2% | 0.5% | 0.70 | 0/6 | 0/6 |
| Soc5. Keeping up with  other children | 66.0% | 2.4% | 0.73 | 0/6 | 0/6 |
| **6-13 years** | | | |  |  |
| Soc1. Getting along with  others | 39.5% | 4.7% | 0.79 | 4/6 | 5/6 |
| Soc2. Others not wanting  to be friends | 43.4% | 0.9% | 0.78 | 2/6 | 1/6 |
| Soc3. Getting teased | 42.4% | 0.7% | 0.76 | 0/6 | 0/6 |
| Soc4. Unable to do things  others can do | 59.3% | 0.8% | 0.69 | 0/6 | 0/6 |
| Soc5. Keeping up with  other children | 67.3% | 4.0% | 0.72 | 0/6 | 0/6 |
| **14-17 years** | | | |  |  |
| Soc1. Getting along with  others | 40.3% | 4.7% | 0.79 | 4/6 | 2/3 |
| Soc2. Others not wanting  to be friends | 51.4% | 0.6% | 0.80 | 2/6 | 1/3 |
| Soc3. Getting teased | 54.0% | 0.6% | 0.77 | 0/6 | 0/3 |
| Soc4. Unable to do things  others can do | 61.1% | 1.3% | 0.75 | 0/6 | 0/3 |
| Soc5. Keeping up with  other children | 61.0% | 3.2% | 0.78 | 0/6 | 0/3 |

Abbreviations: Soc, Social Functioning.

^a^ Correlation of item score with dimension score (i.e., Spearman’s correlation coefficient).

^b^ Number of experts (out of *n* = 6) that chose an item as the best item to represent this dimension.

^c^ Number of parents that chose an item as the best item to represent this dimension. There were *n* = 6 parents with children 2-5 years old, *n* = 6 parents with children 6-13 years old, and *n* = 3 parents for children 14-17 years old (*n* = 12 parents but some parents had children in different age groups so total adds up to more than 12).

# Appendix Table 5D. Summary of Psychometric Analysis and Expert and Parent Opinion – School Functioning

| **Item Description**  *Problems with…* | **Psychometric Analysis** | | | **Expert and Parent Opinion** | |
| --- | --- | --- | --- | --- | --- |
|  | **% Response Ceiling (Never)** | **% Response Floor**  **(Almost always)** | **Internal Consistency**^a^ | **Best item – Experts**^b^ | **Best item – Parents**^c^ |
| **2-5 years** | | | |  |  |
| School3. Keeping up with  schoolwork | 62.5% | 2.6% | N/A^d^ | 6/6 | 6/6 |
| **6-13 years** | | | |  |  |
| School1. Paying attention  in class | 32.7% | 4.6% | 0.90 | 0/6 | 0/6 |
| School2. Forgetting  things | 21.3% | 1.5% | 0.78 | 0/6 | 0/6 |
| School3. Keeping up with  schoolwork | 39.2% | 5.0% | 0.89 | 6/6 | 6/6 |
| **14-17 years** | | | |  |  |
| School1. Paying attention  in class | 32.5% | 4.3% | 0.91 | 0/6 | 0/3 |
| School2. Forgetting  things | 24.4% | 1.2% | 0.79 | 0/6 | 0/3 |
| School3. Keeping up with  schoolwork | 26.5% | 5.4% | 0.89 | 6/6 | 3/3 |

Abbreviations: N/A, not applicable; SchAbs, School Absence.

^a^ Correlation of item score with dimension score (i.e., Spearman’s correlation coefficient).

^b^ Number of experts (out of *n* = 6) that chose an item as the best item to represent this dimension.

^c^ Number of parents that chose an item as the best item to represent this dimension. There were *n* = 6 parents with children 2-5 years old, *n* = 6 parents with children 6-13 years old, and *n* = 3 parents for children 14-17 years old (*n* = 12 parents but some parents had children in different age groups so total adds up to more than 12).

^d^ Only School3 is included in the PedsQL for this dimension for this age group, thus correlation was not applicable.

# Appendix Table 5E. Summary of Psychometric Analysis and Expert and Parent Opinion – School Absence

| **Item Description**  *Problems with…* | **Psychometric Analysis** | | | **Expert and Parent Opinion** | |
| --- | --- | --- | --- | --- | --- |
|  | **% Response Ceiling (Never)** | **% Response Floor**  **(Almost always)** | **Internal Consistency**^a^ | **Best item – Experts**^b^ | **Best item – Parents**^c^ |
| **2-5 years**^d^ | | | |  |  |
| SchAbs1. Missing school  because sick | 58.2% | 0.1% | 0.91 | 6/6 | 6/6 |
| SchAbs2. Missing school  to go to doctor | 74.3% | 0.1% | 0.88 | 0/6 | 0/6 |
| **6-13 years** | | | |  |  |
| SchAbs1. Missing school  because sick | 47.0% | 0.3% | 0.90 | 6/6 | 6/6 |
| SchAbs2. Missing school  to go to doctor | 64.9% | 0.3% | 0.88 | 0/6 | 0/6 |
| **14-17 years** | | | |  |  |
| SchAbs1. Missing school  because sick | 38.3% | 1.2% | 0.91 | 6/6 | 3/3 |
| SchAbs2. Missing school  to go to doctor | 56.3% | 0.5% | 0.88 | 0/6 | 0/3 |

Abbreviations: SchAbs, School Absence.

^a^ Correlation of item score with dimension score (i.e., Spearman’s correlation coefficient).

^b^ Number of experts (out of *n* = 6) that chose an item as the best item to represent this dimension.

^c^ Number of parents that chose an item as the best item to represent this dimension. There were *n* = 6 parents with children 2-5 years old, *n* = 6 parents with children 6-13 years old, and *n* = 3 parents for children 14-17 years old (*n* = 12 parents but some parents had children in different age groups so total adds up to more than 12).

^d^ School Absence items were not asked for children aged 2-3 years in the LSAC, thus psychometric analysis results reflect responses for children aged 4-5 years.

# Appendix Table 6. Summary of Item Performance and Reason for Exclusion/Inclusion

| **Item Description**  *Problems with…* | **Step 1. Reason for Exclusion**^a^ | **Excluded in Step 1** | **Step 2. Summary of Item Performance of Remaining Items** | **Selected for HSCS** |
| --- | --- | --- | --- | --- |
| **Physical Functioning** | | | | |
| Phys1. Walking | Worst performing item according to Rasch criteria for all ages in supplemental analyses (total score 0/3 for 2-5 years, 1/5 for 6-13 years, and 1/3 for 14-17 years) so excluded from HSCS. | X |  |  |
| Phys2. Running |  |  | In supplemental analyses, reasonable fit to Rasch model for age groups 6-13 years (total score 4/5) and 14-17 years (total score 3/3). No physical functioning item fit Rasch model in supplemental analyses for age group 2-5 years.  Larger ceiling effects present for all ages than Phys3.  High internal consistency but lower than Phys3.  Only 1 parent chose as best item to represent the dimension.^b^ |  |
|  |  |  |  |  |
|  |  |  |  |  |
| Phys3. Participating in exercise |  |  | Only item that performed well on all Rasch criteria for the subsample that fit the initial Rasch model for ages 2-5 years and 6-13 years.  In supplemental analyses, reasonable fit to Rasch model for age groups 6-13 years (total score 4/5) and 14-17 years (total score 3/3). No physical functioning item fit Rasch model in supplemental analyses for age group 2-5 years.  Ceiling effects present for all ages, but less severe than Phys2.  Higher internal consistency for all ages than Phys2.  Most experts and parents chose as best item to represent the dimension.^b^ | ✓ |
|  |  |  |  |  |
|  |  |  |  |  |
| Phys4. Lifting something heavy | Poorly fit Rasch model for ages 2-5 years and 6-13 years (total score 0/5) and considered to not properly represent the dimension by research team so omitted from supplemental analyses. | X |  |  |
| Phys5. Taking a bath or shower | Poorly fit Rasch model for all ages (total score 0/5) so item omitted from supplemental analyses. | X |  |  |
| Phys6. Doing chores | Poorly fit Rasch model for all ages (total score 0/5) so item omitted from supplemental analyses. | X |  |  |
| **Pain** | | | | |
| Having hurts or aches | N/A^c^ |  | N/A | ✓ |
| **Fatigue** | | | | |
| Low energy level | N/A^c^ |  | N/A | ✓ |
| **Emotional Functioning** | | | | |
| Emot1. Feeling afraid or scared |  |  | Reasonable fit according to Rasch criteria for age group 2-5 years (total score 4/5) but may exhibit slight misfit for age groups 6-13 years and 14-17 years (total score 2/5).  Larger item spread for age groups 2-5 years and 6-13 years than Emot5.  Larger ceiling effects for age group 14-17 years than Emot5.  High internal consistency for all ages similar to Emot5.  Most experts and parents chose as best item to represent the dimension for age group 2-5 years.^b^ |  |
| Emot2. Feeling sad or blue | Worst performing item according to Rasch criteria for age groups 2-5 years (total score 1/5) and 14-17 years (total score 0/5) so excluded from HSCS. | X |  |  |
| Emot3. Feeling angry |  |  | Among remaining items, Emot3 was the worst performing item based on Rasch criteria for age groups 6-13 years and 14-17 years (total score 1/5) and had lowest internal consistency for all ages so was excluded from HSCS. |  |
| Emot4. Trouble sleeping | Worst performing item according to Rasch criteria for age groups 6-13 years (total score 0/5) so excluded from HSCS. | X |  |  |
| Emot5. Worrying |  |  | Best fit according to Rasch criteria across all age groups (total score 4/5 for 2-5 years and 5/5 for both 6-13 years and 14-17 years).  Larger item spread for age group 14-17 years than Emot1.  Larger ceiling effects for age group 2-4 years than Emot1.  High internal consistency for all ages similar to Emot1.  Most parents chose as best item to represent the dimension for age groups 6-13 years and 14-17 years and one expert chose as best item for all ages.^b^  Health status measurement expert thought Emot5 may better express emotional functioning pathology. | ✓ |
| **Social Functioning** | | | | |
| Soc1. Getting along with others | Poorly fit Rasch model for all ages (total score 0/5) so excluded from HSCS. | X |  |  |
| Soc2. Others not wanting to be friends |  |  | Best fit according to Rasch criteria across all age groups (total score 4/5 for both 2-5 years and 6-13 years and 3/5 for 14-17 years).  Larger item spread for all ages than Soc3.  Ceiling effects present across all ages, but less severe than Soc3.  Higher internal consistency for all ages than Soc3.  Two experts and five parents chose as best item to represent the dimension across age groups.^d^ | ✓ |
| Soc3. Getting teased |  |  | Reasonable fit according to Rasch criteria for age group 2-5 years (total score 4/5) but may exhibit misfit for age groups 6-13 years (total score 1/5) and 14-17 years (total score 2/5).  Larger ceiling effects than Soc2.  High internal consistency for all ages but lower than Soc2.  No experts and parents chose as best item to represent the dimension for any age group. |  |
| Soc4. Unable to do things others can do | Poorly fit Rasch model for all ages (total score 1/5 for 2-5 years and 0/5 for 6-13 years and 14-17 years) so excluded from HSCS. | X |  |  |
| Soc5. Keeping up with other children | Poorly fit Rasch model for all ages (total score 0/5) so excluded from HSCS. | X |  |  |
| **School Functioning** | | | | |
| School1. Paying attention in class | Not a validated item for age group 2-5 years (only School3 included in PedsQL for this age group) and poorly fit Rasch model for age groups 6-13 years (total score 0/5) and 14-17 years (total score 2/5) so excluded from HSCS. | X |  |  |
| School2. Forgetting things | Not a validated item for age group 2-5 years (only School3 included in PedsQL for this age group) and poorly fit Rasch model for age groups 6-13 years and 14-17 years (total score 0/5) so excluded from HSCS. | X |  |  |
| School3. Keeping up with schoolwork |  |  | Only item included in PedsQL for age group 2-5 years.  Exhibited better fit according to Rasch criteria for age group 14-17 years (total score 4/5).  Largest item spread.  Exhibited some ceiling effects and was largest for age group 2-5 years.  High internal consistency.  All parents and experts chose as best item to represent the dimension for all ages.^d^ | ✓ |
| **School Absence**^e^ | | | | |
| SchAbs1. Missing school because sick |  |  | Exhibited better fit according to Rasch criteria for all ages (total score 2/5 for 2-5 years, 4/5 for 6-13 years, and 3/5 for 14-17 years).  Large item spread.  Highest internal consistency.  Ceiling effects present across all ages, but less severe than SchAbs2.  All parents and experts chose as best item to represent the dimension for all ages.^d^ | ✓ |
| SchAbs2. Missing school to go to doctor | Poorly fit Rasch model for all ages (total score 0/5) so excluded from HSCS. | X |  |  |

Abbreviations: HSCS, health state classification system; N/A, not applicable.

^a^ In general, any item that performed poorly across all subsamples (i.e., total score 0/5) or was worst fitting item in any age group (i.e., lowest total score) was excluded in Step 1.

^b^ One of the items labeled as “candidate for best question” when collecting expert and parent opinions. See Appendix B for more details.

^c^ Single-item dimensions were not evaluated empirically in item selection since they only included one item.

^d^ Labeled as “best question” when collecting expert and parent opinions. See Appendix B for more details.

^e^ None of the subsamples could be fitted to the overall Rasch model, thus Rasch item-specific results are for Rasch models with statistically significant item-trait interaction $\chi^{2}$ statistics.

# Appendix Table 7A. Correlation Between Dimensions for Age Group 2-5 Years

| **Dimension** | **Physical Functioning** (“Participating in exercise”) | **Pain**  (“Having hurts or aches”) | **Fatigue**  (“Low energy level”) | **Emotional Functioning**  (“Worrying”) | **Social Functioning**  (“Others not wanting to be friends”) | **School Functioning**  (“Keeping up with schoolwork”) | **School Absence**  (“Missing school because sick”) |
| --- | --- | --- | --- | --- | --- | --- | --- |
| **Physical Functioning**  (“Participating in exercise”) | 1.00 | – | – | – | – | – | – |
| **Pain**  (“Having hurts or aches”) | 0.17 | 1.00 | – | – | – | – | – |
| **Fatigue**  (“Low energy level”) | 0.21 | 0.40 | 1.00 | – | – | – | – |
| **Emotional Functioning**  (“Worrying”) | 0.18 | 0.29 | 0.32 | 1.00 | – | – | – |
| **Social Functioning**  (“Others not wanting to be friends”) | 0.23 | 0.24 | 0.24 | 0.32 | 1.00 | – | – |
| **School Functioning**  (“Keeping up with schoolwork”) | 0.30 | 0.16 | 0.19 | 0.20 | 0.32 | 1.00 | – |
| **School Absence**  (“Missing school because sick”) | 0.14 | 0.23 | 0.22 | 0.17 | 0.15 | 0.20 | 1.00 |

# Appendix Table 7B. Correlation Between Dimensions for Age Group 6-13 Years

| **Dimension** | **Physical Functioning** (“Participating in exercise”) | **Pain**  (“Having hurts or aches”) | **Fatigue**  (“Low energy level”) | **Emotional Functioning**  (“Worrying”) | **Social Functioning**  (“Others not wanting to be friends”) | **School Functioning**  (“Keeping up with schoolwork”) | **School Absence**  (“Missing school because sick”) |
| --- | --- | --- | --- | --- | --- | --- | --- |
| **Physical Functioning**  (“Participating in exercise”) | 1.00 | – | – | – | – | – | – |
| **Pain**  (“Having hurts or aches”) | 0.23 | 1.00 | – | – | – | – | – |
| **Fatigue**  (“Low energy level”) | 0.32 | 0.45 | 1.00 | – | – | – | – |
| **Emotional Functioning**  (“Worrying”) | 0.18 | 0.31 | 0.32 | 1.00 | – | – | – |
| **Social Functioning**  (“Others not wanting to be friends”) | 0.21 | 0.24 | 0.27 | 0.37 | 1.00 | – | – |
| **School Functioning**  (“Keeping up with schoolwork”) | 0.35 | 0.18 | 0.25 | 0.24 | 0.29 | 1.00 | – |
| **School Absence**  (“Missing school because sick”) | 0.22 | 0.33 | 0.31 | 0.22 | 0.19 | 0.24 | 1.00 |

# Appendix Table 7C. Correlation Between Dimensions for Age Group 14-17 Years

| **Dimension** | **Physical Functioning** (“Participating in exercise”) | **Pain**  (“Having hurts or aches”) | **Fatigue**  (“Low energy level”) | **Emotional Functioning**  (“Worrying”) | **Social Functioning**  (“Others not wanting to be friends”) | **School Functioning**  (“Keeping up with schoolwork”) | **School Absence**  (“Missing school because sick”) |
| --- | --- | --- | --- | --- | --- | --- | --- |
| **Physical Functioning**  (“Participating in exercise”) | 1.00 | – | – | – | – | – | – |
| **Pain**  (“Having hurts or aches”) | 0.32 | 1.00 | – | – | – | – | – |
| **Fatigue**  (“Low energy level”) | 0.34 | 0.55 | 1.00 | – | – | – | – |
| **Emotional Functioning**  (“Worrying”) | 0.23 | 0.35 | 0.45 | 1.00 | – | – | – |
| **Social Functioning**  (“Others not wanting to be friends”) | 0.22 | 0.26 | 0.34 | 0.44 | 1.00 | – | – |
| **School Functioning**  (“Keeping up with schoolwork”) | 0.29 | 0.23 | 0.32 | 0.30 | 0.27 | 1.00 | – |
| **School Absence**  (“Missing school because sick”) | 0.26 | 0.42 | 0.44 | 0.36 | 0.28 | 0.38 | 1.00 |

# References

1. Pallant JF, Tennant A. An introduction to the Rasch measurement model: an example using the Hospital Anxiety and Depression Scale (HADS). Br J Clin Psychol. 2007;46(Pt 1):1-18.

2. Tennant A, Conaghan PG. The Rasch measurement model in rheumatology: what is it and why use it? When should it be applied, and what should one look for in a Rasch paper? Arthritis Rheum. 2007;57(8):1358-62.

3. Costa DS, Aaronson NK, Fayers PM, Grimison PS, Janda M, Pallant JF, et al. Deriving a preference-based utility measure for cancer patients from the European Organisation for the Research and Treatment of Cancer's Quality of Life Questionnaire C30: a confirmatory versus exploratory approach. Patient Relat Outcome Meas. 2014;5:119-29.

4. Jörngården A, Wettergen L, von Essen L. Measuring health-related quality of life in adolescents and young adults: Swedish normative data for the SF-36 and the HADS, and the influence of age, gender, and method of administration. Health Qual Life Outcomes. 2006;4:91.

5. Arrington-Sanders R, Yi MS, Tsevat J, Wilmott RW, Mrus JM, Britto MT. Gender differences in health-related quality of life of adolescents with cystic fibrosis. Health Qual Life Outcomes. 2006;4:5.

6. Brazier JE, Mulhern BJ, Bjorner JB, Gandek B, Rowen D, Alonso J, et al. Developing a New Version of the SF-6D Health State Classification System From the SF-36v2: SF-6Dv2. Med Care. 2020;58(6):557-65.

7. Karami H, Salmani Nodoushan MA. Differential Item Functioning (DIF): Current problems and future directions. International Journal of Language Studies. 2011;5:133-42.

8. Hagquist C, Andrich D. Recent advances in analysis of differential item functioning in health research using the Rasch model. Health and Quality of Life Outcomes. 2017;15(1):181.

9. King MT, Costa DS, Aaronson NK, Brazier JE, Cella DF, Fayers PM, et al. QLU-C10D: a health state classification system for a multi-attribute utility measure based on the EORTC QLQ-C30. Qual Life Res. 2016;25(3):625-36.
